# A three-stage machine learning and inference approach for educational data

**DOI:** 10.1038/s41598-025-89394-2

**Published:** 2025-04-04

**Authors:** Ting Da

**Affiliations:** National Engineering Research Center of Cyberlearning and Intelligent Technology, Beijing Normal University, Beijing, China

**Keywords:** Machine learning, Causal inference, OLS regression, Instrumental variable (IV), LASSO, Engineering, Mathematics and computing

## Abstract

A central task in educational studies is to uncover factors that drive a student’s academic performance. While existing studies have utilized meticulous regression designs, it is challenging to select appropriate controls. Machine learning, however, offers a solution whereby the entire variable set can be inspected and filtered by different optimization schemes. In that light, this paper adopts a three-stage framework to analyze and discover potentially latent causal relationships from an open dataset from UCI. In the first stage, machine learning methods are employed to select candidate variables that are closely associated with student grades, and then a “post-double-selection” process is implemented to select the set of control variables. In the final stage, three case studies are conducted to illustrate the effectiveness of the three-stage design. The model pipeline is suitable for situations where there is only minimal prior knowledge available to address a potentially causal research question.

## Introduction

What are the driving forces of a student’s course performance? To what extent does the family influence the student? How does past performance affect current behavior? Such questions have been central in educational studies and have sparked a wide spectrum of analysis for decades.

Conventional educational studies typically involve meticulous sampling and collection of student-level characteristics from prior knowledge. The theme is to test various hypotheses of potential explanatory variables, through linear regressions or structural equation models (SEMs)^1^. In recent years, machine learning algorithms have provided an alternative whereby researchers are able to forecast the future performance of a student based on past observations.

While these two streams of methods have been employed separately for years, cutting-edge research is now devoted to the synthesis of the two sets of tools. The most prominent example is the least absolute shrinkage and selection operator (LASSO)^2^, which was devised to strike a balance between prediction accuracy and the size of the feature space. The algorithm outputs a condensed feature set and was initially used to remedy the curse of dimensionality emphasized in machine learning, which detects poor out-of-sample predictions given a large set of features. Currently, due to its interpretability and speed of implementation, LASSO has also been adopted in many fields of social science to guide the process of feature selection.

It is worth mentioning that linear regression and SEMs also rely upon a relatively small set of features, and the literature in education has mostly used prior knowledge to determine which variables to include in the model. However, machine learning algorithms offer another perspective, where no prior knowledge is required and meaningless features are filtered out purely based on the optimization process of the algorithm.

In that light, a three-stage procedure is proposed in this paper to demonstrate how machine learning could be coupled with linear regression and instrumental variables (IVs) to make statistical or even causal inferences in education. This paper investigates student-level course performance data provided by^3^ (the UCI dataset). The dataset involves hundreds of students and a host of student-level characteristics, such as sex, age, family background, and the intention of pursuing a higher degree. While these characteristics are observed cross-sectionally, the main outcome variables (course grades) are recorded for three consecutive periods. Past researchers have focused on predicting future course performance based on historical values and student-level characteristics. However, our analysis stresses the new approach of inference, where the sets of explanatory and control variables are chosen by algorithms instead of purely from prior knowledge.

Specifically, in stage 1, this work first expands the original feature space by including interacting features and taking the squares of the numerical variables. The interactions are essentially moderators, and the squared terms capture increasing or diminishing effects.

The enriched feature set is then passed to three prevailing machine learning algorithms—LASSO logistic, random forest^4^ and LASSO regression—to obtain a subset of variables that are correlated with course grades. Note that each method of choice encodes a different feature selection criterion; hence, comparing and taking the union of the filtered feature spaces of the three models can reduce the feature selection bias due to the similar optimization schemes.

Inspecting the condensed feature set, in stage 2, three representative explanatory variables is selected and a post-double-selection process is implemented to find the set of control variables associated with each explanatory variable. This technique was initially introduced in econometrics and has now become popular in big data inference. The merit of this process is its robustness to imperfect variable selection.

Coupling each explanatory variable with the control variables suggested by stage 2, this paper performs three sets of linear regressions with potential IVs to draw statistical or even causal inferences. However, a major threat to the regression is omitted variable bias due to unobserved confounding factors—most notably, student ability. Given no appropriate proxy for it, this manuscript utilizes recent advances in econometrics to conduct a host of sensitivity analyses to check the stability of the point estimates of the explanatory variables when the confounder is seen to have an impact at different levels of magnitude than existing variables in the regression.

It is found that the educational level of mothers outweighs that of fathers in improving students’ grades. If a student aspires to pursue a higher education, performance will be better. In addition, an IV regression indicates that greater class absence leads to poorer course performance.

The rest of the paper is organized as follows. Section "Literature review" surveys the literature on the two strands of methods in educational studies. Section "Data" describes the data. Section "Three-stage Machine learning and inference approach" carries out the three-stage modeling process. In particular, Section "Stage 1: machine learning"–stage 1—demonstrates how machine learning methods are used to screen the variable space and identify potential explanatory variables. Section "Stage 2: post-double-LASSO for the control variables" performs a post-double-selection process to select the set of control variables. Section "Stage 3: inference based on OLS regression" is the last stage, in which three case studies are conducted and the control variables are guided by the results from stage 2. Section "Concluding remarks" offers concluding remarks.

## Literature review

Traditionally, many empirical analyses in education have focused on discovering causal relationships from a determinant factor to some outcome of interest or causality where multiple factors work in a structural manner. For example, in researches on cognition-related aspects, Adekitan and Noma-Osaghae^5^ analyzed the relationship between the cognitive admission entry requirements and the academic performance of students in their first year. Fischer et al.^6^ examined non-cognitive factors that could predict secondary school grades and investigated reasons that would prompt female students to outperform their male peers. Some studies focused on parental background. For instance, Pandaya^7^ demonstrated a consequential interplay among parental qualification, profession, economic status, and mathematical achievement of their children. On the contribution of teachers, Gustavsen^8^ suggested that teacher-rated social skills have a long-lasting influence on boys’ and girls’ academic achievement over years, and the effect is heterogeneous across subjects. And in^9^, the authors estimated the impact of teacher absences on academic achievement of students in elementary schools. Moreover, there are also recent international evidence (see^10^) suggesting that the differences between countries in the way that the science and mathematics curriculum is constructed may account for the differences in student performance.

In terms of monetary outcomes, in the seminal paper, Card^11^ investigated monetary return relative to years of education, where the causal conclusion is drawn using quarter of birth as an IV. On a broader set of socio-economic outcomes, for example, Siddiky and Akter^12^ examined educational factors that influence the choice of profession and identified strategies of career preparation.

Related to this paper, Gottfried^13^ showed stark difference in the grades between students with high rates of excused and unexcused absences. Specifically, a larger fraction of excused absences are associated with higher reading and math test scores. Conversely, a higher proportion of unexcused absences induces academic risk, particularly in math. In another research, Gershenson et al.^14^ confirmed that in general, absences are associated with statistically significant deterioration in academic achievement.

In addition, extensive literature has utilized SEM to study the interplay of various driving forces of a student’s academic performance. For instance, Núñez^15^ suggested a recursive relationship between secondary school students’ achievement and their perceptions of parental involvement in homework. Cooper et al.^16^ indicated that positive student norms, higher student ability, and positive parent attitudes toward homework were all related to greater parent facilitation. Moreover, Cox and Williams^17^ illustrated the positive roles of perceived competence, autonomy, and mastery climate in physical education. And^18^ presented that parent involvement in school is associated with better academic outcomes and social competency in children.

Regarding empirical methodology, recent decades have witnessed monumental breakthroughs in machine learning and the data available for academic research. As most machine algorithms target high-dimensional datasets (where the number of features may be thousands, tens of thousands, or more), for which it is difficult to interpret the features, a wide spectrum of methods have been developed to extract a low-dimensional subset of features that captures key information contained in the entire feature space. Many of these classical algorithms are summarized in^19^. Among them, the two strands of methods based on regularization—e.g., LASSO by^2^, Ridge by^20^, and Elastic Net by^21^—and trees—e.g., decision trees by^22^ and random forests by^4^—have been shown to be effective and interpretive in a generic problem context and therefore are popular in many academic fields. Early researchers attempted to make inferences on unknown underlying parameters directly from the estimated coefficients of these dimension-reduction models. However, rigorous analysis of the statistical properties of these methods by^23^ showed that this process often leads to biased conclusions.

To address this issue, a natural solution is to use machine learning algorithms and regressions separately and let each set of models do what it is best at. This general modeling pipeline has been adopted in many areas of social science, for example, economics, education, etc. This intuitive approach, nevertheless, often suffers from another crucial source of bias induced by imperfect variable selection, as proven in the literature of double machine learning; see^24^ and^25^ for details. The idea is that some confounding variables may have small but nonzero effects, and it is therefore likely that the dimension-reduction algorithms will overlook these potential confounders. Such ignorance leads to omitted variable bias, which contaminates estimation and inference in the follow-up regression.

Given the source of bias, Belloni et al.^24^ proposed a “post-double-selection” procedure to combine the two types of methodologies in a discretionary manner. It was proven that the procedure is robust to imperfect variable selection as well as non-Gaussian distribution and heteroskedasticity of the error term in the model. Notably, their results are also applicable to a partially linear regression setup, which allows for flexible functional forms of the control variables. Ahrens et al.^26^ provided a practical implementation of these methods.

More broadly speaking, many other researchers have further studied how to synthesize machine learning and causal inference. Wager and Athey^27^ presented a nonparametric causal forest for estimating heterogeneous treatment effects that extends Breiman’s widely used random forest algorithm. Athey and Imbens^28^ highlighted that newly developed methods at the intersection of machine learning and econometrics typically perform better than either off-the-shelf individual methods in many contexts. Athey^29^ provided an assessment of the early contributions of machine learning to economics, as well as predictions about its future contributions.

It is also worth mentioning that a great majority of studies especially in the machine learning community have focused purely on the prediction of student performance without attempting to draw causal statements. For example, Hasib et al.^30^ demonstrated the high predictive power of machine learning on the academic outcomes of secondary school students. Xu et al.^31^ targeted at predicting students’ accomplishment in degree programs. And^32^ reviewed 70 papers regarding different modern techniques that had been widely applied for performance prediction.

Another approach to educational modeling involves networks, particularly Bayesian networks^33,34^. In Bayesian networks, nodes represent variables, and domain knowledge is used to construct directed edges that indicate potential causal relationships. Data is then utilized to estimate the conditional probabilities given the parent nodes. With the resulting probability distribution, researchers can explore “what if” scenarios. However, the validity of these networks critically depends on correctly specifying all relevant latent variables, as any omission can lead to confounding issues and biased estimates. Additionally, Bayesian networks are not computationally efficient for large-scale networks. As the number of variables increases, the complexity of the network structure grows nearly exponentially, making probability computations increasingly intractable.

Lastly, one extra synthesis between education and machine learning lies in dynamic treatment regimes (DTRs)^35–39^ as DTRs could enable personalized, adaptive interventions that evolve with students’ needs. Like in healthcare, challenges such as unobserved confounders and time-varying effects are prevalent in educational data. Machine learning methods, particularly reinforcement learning^40^, address these challenges by optimizing interventions using large, observational datasets. Techniques like Direct Augmented V-Learning (DAV-Learning) and Safe Augmented V-Learning (SAV-Learning) can model dynamic interactions between students’ learning progress and intervention effects. These methods enable two-way personalization, tailoring strategies based on both student and teacher characteristics.

## Data

The data used in this paper come from^3^ and are maintained by the University of California—Irvine (the UCI dataset). These publicly available data contain two cross-sectional dataframes that record student-level variables and test grades for Math and Portuguese. The dataset was obtained using school reports and questionnaires. Standard demographic, social, and school related features were collected, which provide opportunities for analysis from correlational, causal, and machine-learning types of researches. However, the students in the two datasets are anonymous, which precludes merging of the samples.

**Fig. 1:**
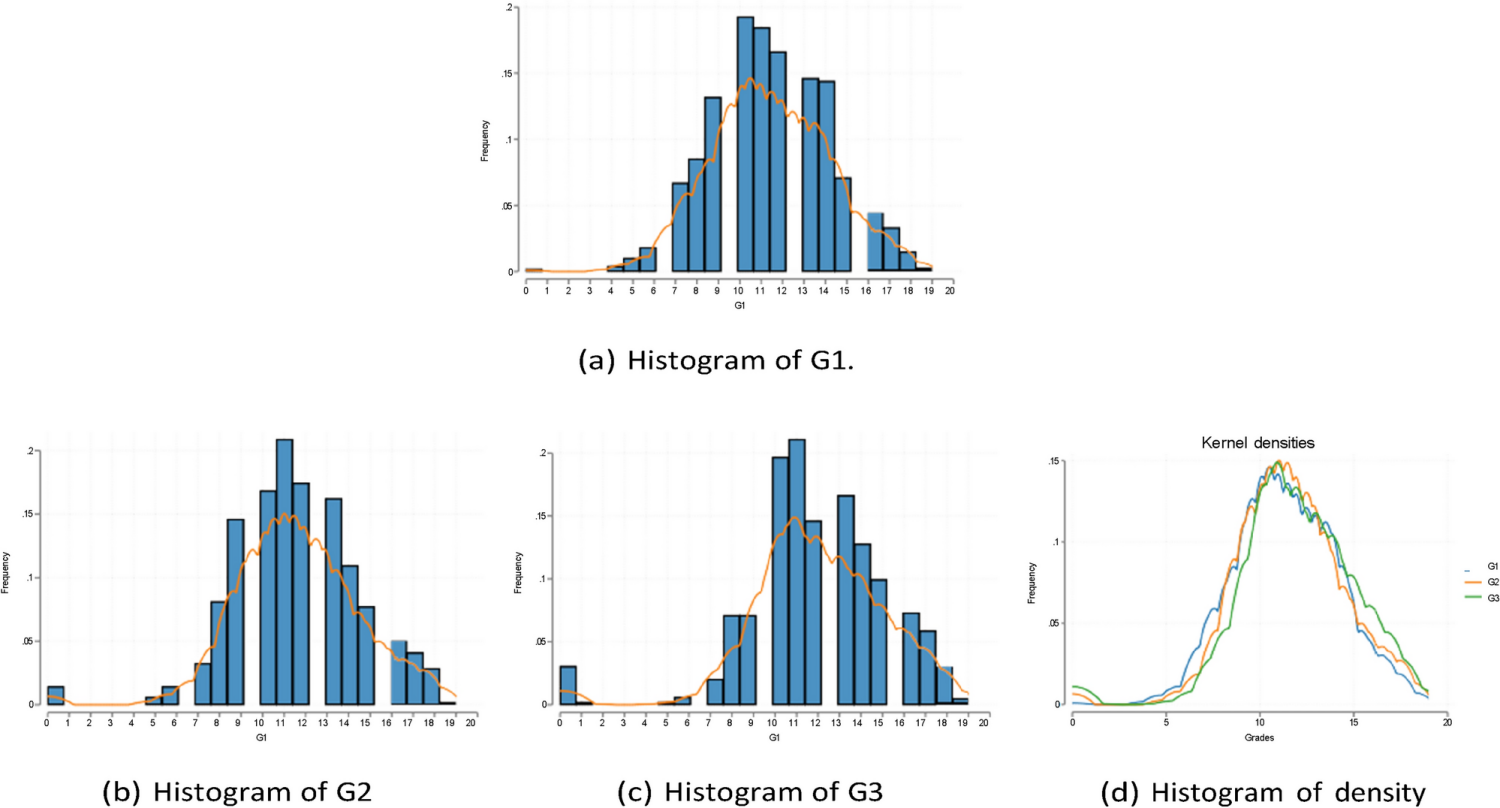
Histogram of G1, G2, and G3 in Portuguese.

**Fig. 2:**
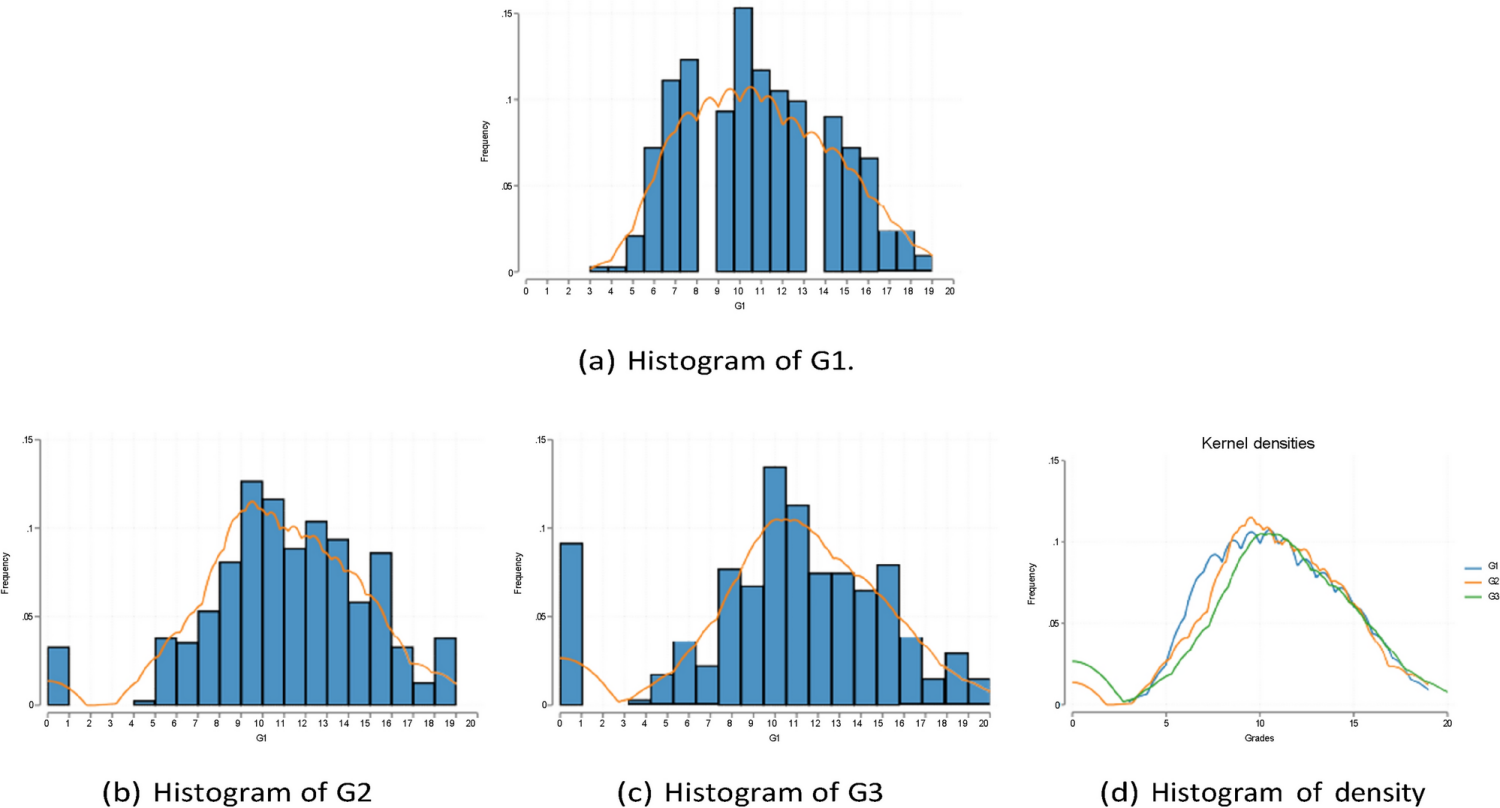
Histogram of G1, G2, and G3 in Math.

Summary statistics for all variables are shown in Tables 1 and 2. The Math and Portuguese samples each contain 395 and 649 observations. Among them, the categorical features include the reason and choice of school, sex, address (urban vs. rural), family size, parents’ marriage status (divorced or not), parents’ educational level, guardian, academic support from family and school, whether the student is working at a paid job, and frequency of engaging in extracurricular activities. Note that the mean shown in the table for the one-hot version of these categorical variables are indeed the proportion of taking the value one. The standard deviation measures the variability of these binary encodings. The main outcomes of interest are the course grades for the two subjects in the three observational periods. For a complete list of variable descriptions and variable names, see Table 1 in Cortez and Silva^3^. Histograms of G1 G2 G3 (i.e., grades for the three consecutive periods) in Math and Portuguese are shown in Figs. 1 and 2.

**Table 1:** Summary statistics for math.

	N	Mean	Std. dev.	10th	90th
Age	395	16.70	1.28	15.00	18.00
Gender (Female)	395	0.53	0.50	0.00	1.00
School (GP)	395	0.88	0.32	0.00	1.00
Higher (Yes)	395	0.95	0.22	1.00	1.00
Address (Urban)	395	0.78	0.42	0.00	1.00
Absences	395	5.71	8.00	0.00	14.00
Romantic (Yes)	395	0.33	0.47	0.00	1.00
Mother’s edu. (none)	395	0.01	0.09	0.00	0.00
Mother’s edu. (4th grade)	395	0.15	0.36	0.00	1.00
Mother’s edu. (5th to 9th grade)	395	0.26	0.44	0.00	1.00
Mother’s edu. (secondary education)	395	0.25	0.43	0.00	1.00
Mother’s edu. (higher education)	395	0.33	0.47	0.00	1.00
Father’s edu. (none)	395	0.01	0.07	0.00	0.00
Father’s edu. (4th grade)	395	0.21	0.41	0.00	1.00
Father’s edu. (5th to 9th grade)	395	0.29	0.45	0.00	1.00
Father’s edu. (secondary education)	395	0.25	0.44	0.00	1.00
Father’s edu. (higher education)	395	0.24	0.43	0.00	1.00
Studytime	395	2.04	0.84	1.00	3.00
Mother’s job (teacher)	395	0.15	0.35	0.00	1.00
Mother’s job (health)	395	0.09	0.28	0.00	0.00
Mother’s job (at home)	395	0.15	0.36	0.00	1.00
Mother’s job (services)	395	0.26	0.44	0.00	1.00
Mother’s job (other)	395	0.36	0.48	0.00	1.00
Father’s job (teacher)	395	0.07	0.26	0.00	0.00
Father’s job (health)	395	0.05	0.21	0.00	0.00
Father’s job (at home)	395	0.05	0.22	0.00	0.00
Father’s job (services)	395	0.28	0.45	0.00	1.00
Father’s job (other)	395	0.55	0.50	0.00	1.00
Reason (home)	395	0.28	0.45	0.00	1.00
Reason (reputation)	395	0.27	0.44	0.00	1.00
Reason (course)	395	0.37	0.48	0.00	1.00
Reason (other)	395	0.09	0.29	0.00	0.00

**Table 2:** Summary statistics for portuguese.

	N	Mean	Std. dev.	10th	90th
Age	649	16.74	1.22	15.00	18.00
Gender (Female)	649	0.59	0.49	0.00	1.00
School (GP)	649	0.65	0.48	0.00	1.00
Higher (Yes)	649	0.89	0.31	0.00	1.00
Address (Urban)	649	0.70	0.46	0.00	1.00
Absences	649	3.66	4.64	0.00	10.00
Romantic (Yes)	649	0.37	0.48	0.00	1.00
Mother’s edu. (none)	649	0.01	0.10	0.00	0.00
Mother’s edu. (4th grade)	649	0.22	0.41	0.00	1.00
Mother’s edu. (5th to 9th grade)	649	0.29	0.45	0.00	1.00
Mother’s edu. (secondary education)	649	0.21	0.41	0.00	1.00
Mother’s edu. (higher education)	649	0.27	0.44	0.00	1.00
Father’s edu. (none)	649	0.01	0.10	0.00	0.00
Father’s edu. (4th grade)	649	0.27	0.44	0.00	1.00
Father’s edu. (5th to 9th grade)	649	0.32	0.47	0.00	1.00
Father’s edu. (secondary education)	649	0.20	0.40	0.00	1.00
Father’s edu. (higher education)	649	0.20	0.40	0.00	1.00
Studytime	649	1.93	0.83	1.00	3.00
Mother’s job (teacher)	649	0.11	0.31	0.00	1.00
Mother’s job (health)	649	0.07	0.26	0.00	0.00
Mother’s job (at home)	649	0.21	0.41	0.00	1.00
Mother’s job (services)	649	0.21	0.41	0.00	1.00
Mother’s job (other)	649	0.40	0.49	0.00	1.00
Father’s job (teacher)	649	0.06	0.23	0.00	0.00
Father’s job (health)	649	0.04	0.19	0.00	0.00
Father’s job (at home)	649	0.06	0.25	0.00	0.00
Father’s job (services)	649	0.28	0.45	0.00	1.00
Father’s job (other)	649	0.57	0.50	0.00	1.00
Reason (home)	649	0.23	0.42	0.00	1.00
Reason (reputation)	649	0.22	0.41	0.00	1.00
Reason (course)	649	0.44	0.50	0.00	1.00
Reason (other)	649	0.11	0.31	0.00	1.00

**Fig. 3:**
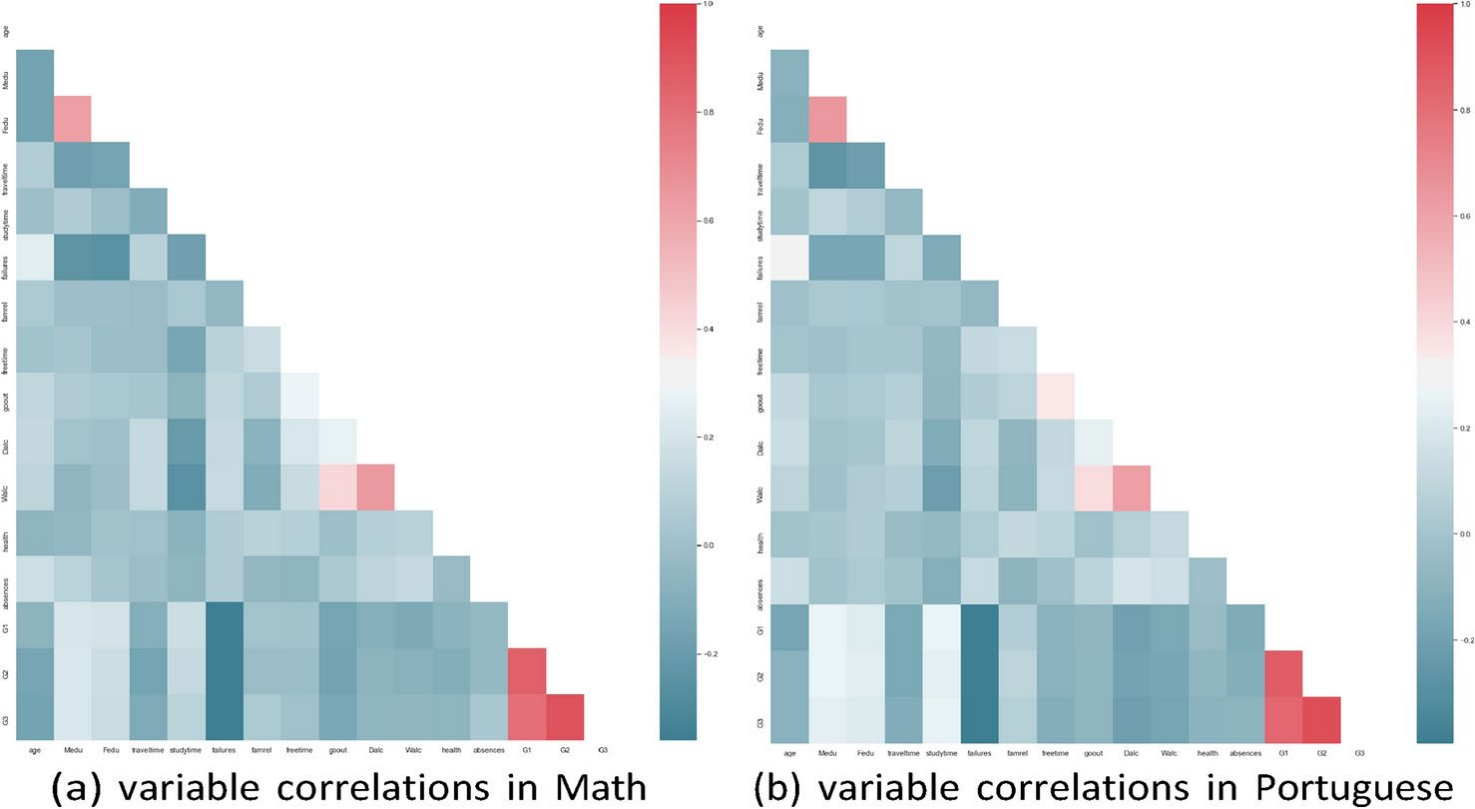
: Variable correlations in two disciplines.

Figure 3 shows the heatmaps of the correlations among variables for Math and Portuguese. Note that the categorical variables inspected here are not in the one-hot form. Instead, they are encoded in the ordinal order of 1, 2, 3, 4, 5, etc, to show the intensity in the response in an increasing strength. For a complete correspondence on the encoding, please refer to the Data Availability section.

The two heatmaps demonstrate that the parents’ educational backgrounds are highly correlated, and course grades are correlated due to their time series nature. In addition, study time has a negative correlation with the frequency of drinking alcohol, and past failures are negatively correlated with current course performance.

Since most variables have nontrivial correlations with others, in the regressions conducted in the third stage, only a subset of the control variables indicated by post- double-LASSO is selected to avoid unstable estimation due to collinearity.

## Three-stage machine learning and inference approach

With the collection of student-level characteristics, there exists a range of machine learning algorithms that could make decent predictions based on the past performance of a student. For example, Cortez and Silva^3^ applied a basic neural network, support vector machine, random forest, etc., to achieve a classification accuracy as high as approximately 90% in binary outcome and a root-mean-square error as low as 1.32 in the Portuguese data. However, machine learning algorithms alone are not capable of making statistical inferences regarding the unknown population relationship between a factor of interest (e.g., parents’ education) and the academic outcome of the student. Therefore, this work adopts a three-stage pipeline that aims to provide a generic approach to making inferences in educational data.

**Fig. 4:**
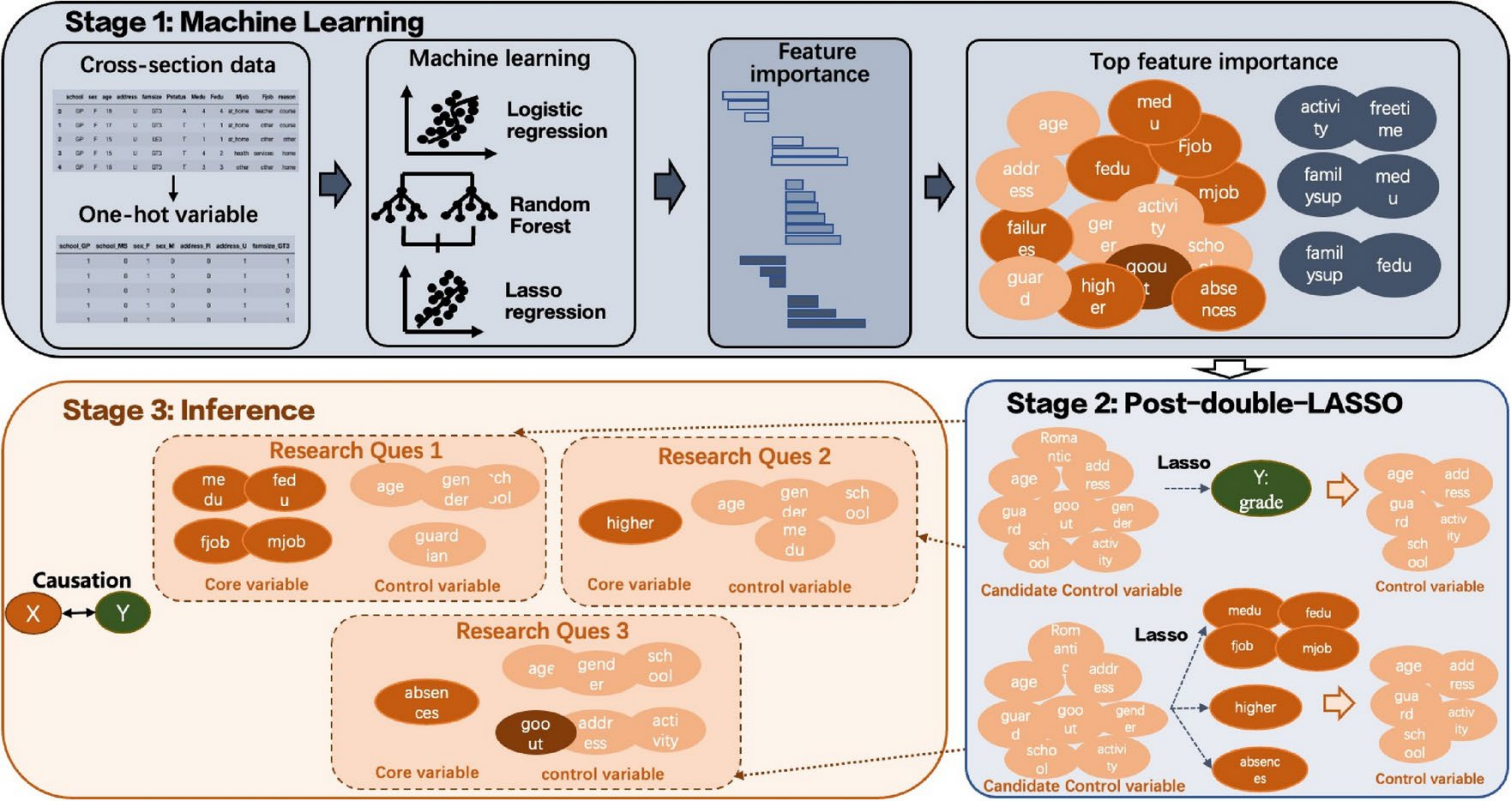
Three-stage machine learning and inference approach.

To put it simply, in the first stage, LASSO logistic regression, random forest, and LASSO regression are implemented on an expanded feature set to obtain a short list of explanatory variables of interest. In the second stage, for each explanatory variable, a post-double-selection process as introduced in^41^ is conducted to obtain a set of potential control variables. The last stage uses linear regression and IVs to draw a statistical and potential causal inference. The paper also demonstrates how recent advances in econometrics could be used to perform sensitivity analysis on the estimated linear regression coefficients under omitted variable bias. Figure 4 outlines the three-stage approach.

On the one hand, the purpose of machine learning is to make predictions, so the smallest error (e.g., mean squared error) is chosen to improve the prediction performance. However, our purpose is not only to predict but also to explain the causal relationships of variables in reality. Machine learning algorithms can flexibly choose a functional form to fit the data to obtain better predictions. Therefore, the more features there are in the machine learning model, the more accurately the results can be predicted. In this way, machine learning algorithms are designed to become increasingly complex. The prediction method of machine learning does not consider the consistency and unbiased characteristics of the estimated coefficients. It simply chooses to trade bias for smaller loss to improve prediction performance.

### Stage 1: machine learning

The target of the first stage is to examine all variables and select a subset of explanatory variables for further inspection in the next two stages. Note that this manuscript differs from^3^ in two ways. First, this work considers the interactions among variables and their squares, while in^3^, only variables in their original forms are inspected, which leads to moderating and diminishing effects being overlooked in the analysis. Second, our choice of machine learning algorithms differs from theirs, as this paper utilizes methods that perform well in dimension reduction instead of prediction accuracy.

That said, this manuscript first expands the set of variables in their original forms by including meaningful interactions of two variables with the student’s age and the squares of the original variables. Note that the one-hot version of the categorical variables is used in the interaction.

Table 3 shows all interactions included. The first seven rows examine whether factors such as the choice of guardian, parenting style, and financial support provider further enhance students’ academic outcomes relative to the time they spend studying. The second row builds on this analysis by exploring the potential moderating effect of students’ health status. Rows 8 to 10 focus on the influence of family education as a moderating factor on students’ academic performance. Rows 11 to 13 investigate the impact of different extracurricular activities, not only in terms of their type but also the amount of time students dedicate to each. Finally, the last four rows analyze the potential heterogeneous effects of engaging in romantic relationships, considering students’ demographic backgrounds and time allocation patterns.

**Table 3:** Interaction variables.

	Vaiable 1	Vaiable 2
1	Guardian (fa/mo)	Studytime (1/2/3/4)
2	Health (1/2/3/4/5 )	Studytime (1/2/3/4)
3	Guardian (fa/mo)	Age
4	Pstatus (T/A)	Famrel (1/2/3/4/5)
5	Paid (Y/N)	Studytime (1/2/3/4)
6	Famsup (Y/N)	Fjob (teacher/health/services/at home/other)
7	Schoolsup (Y/N)	Studytime (1/2/3/4)
8	Higher (Y/N)	Mjob (teacher/health/services/at home/other)
9	Higher (Y/N)	Medu (0/1/2/3/4)
10	Higher (Y/N)	Fedu (0/1/2/3/4)
11	Freetime (1/2/3/4/5)	Goout (1/2/3/4/5)
12	Freetime (1/2/3/4/5)	Activities (Yes/No)
13	Freetime (1/2/3/4/5)	Internet (Yes/No)
14	Romantic (Y/N)	Sex (F/M)
15	Romantic (Y/N)	Age
16	Romantic (Y/N)	Studytime (1/2/3/4)
17	Romantic (Y/N)	Freetime (1/2/3/4)

To reduce the bias that may arise due to similar optimization procedures, the study implements the LASSO logistic model, random forest, and LASSO regression to compress the enriched feature space. Specifically, the LASSO logistic model adds an *l*1 penalty term to the usual logistic objective function that involves the sigmoid transformation. The random forest considers Gini impurity, which measures the relative frequency of misclassification. These two methods view the outcome variable (i.e., course grade) as categorical. And to determine the robustness of the results, the grades are converted into classes in two ways—(i) binary: P/F for passing and failure, and (ii) multiclass: A (≥ 16), B (≥ 14), C (≥ 12), D (≥ 10), and E (< 10).

While other classification algorithms are widely employed in various fields, for our purposes—balancing dimension reduction, interpretability, and effective information utilization—LASSO logistic regression and random forests are the most suitable choices. For example, decision trees could be considered, but their feature selection mechanism is inherently incorporated into random forests, contributing little additional information to the ensemble. Similarly, support vector machines may offer an alternative, but they lack an intuitive framework for assessing the relative importance of selected features. Finally, neural networks typically require substantially larger datasets than ours and lack a transparent method for determining variable importance, making them unsuitable for our proposed methodology.

In contrast, LASSO regression treats course grades as numerical, and its loss function is the mean squared error with an *l*_1_ penalty attached. A similar popular alternative to LASSO, designed for numerical outcome variables, is ridge regression. However, in our context, ridge regression is less suitable because it merely shrinks the coefficients of unimportant variables toward zero without eliminating them entirely. As a result, it does not effectively reduce the feature space, which is a critical requirement for the second and third stages of the proposed pipeline.

Table 4 shows the top ten features with the highest importance determined by each algorithm. In general, this first-stage result provides guidance on which variables could potentially be influential explanatory variables when minimal knowledge is available. In this paper, three variables that are highlighted multiple times by different algorithms are intentionally selected from the table to conduct further analysis in the following two stages. The reason for this choice lies in the different types of endogeneity that one might encounter in a generic educational dataset, which places the pipeline in a more generic setting.

Specifically, the three sets of variables and their related research questions are as follows.

1. Between the parents’ occupation and educational background, which factor is more important?
2. Does the aspiration to pursue a higher degree lead to higher course grades?
3. Does class absence substantially worsen grades?

**Table 4:** Stage 1: variables of top 10 feature importance.

Methods	Grade3	Math	Portuguese
Lasso logistic Reg	Classes (A-E)	1. freetime1 goout2	1. famsup yes Fedu2
		2. Fjob teacher	2. freetime2
		3. failures0	3. goout2
		4. health4 studytime3	4. health2 studytime4
		5. higher yes Fjob teacher	5. freetime4 goout3
		6. health4 studytime2	6. romantic yes sexF
		7. paid yes	7. Medu1
		8. freetime4 goout3	8. schoolsup yes
		9. Guard fa studytime1	9. higher no
		10. schoolsup yes	10. goout1
Lasso Logistic Reg	Classes(P/F)	1. schoolsup yes studytime1	1. failures0
		2. freetime1 goout2	2. freetime1 goout2
		3. failures0	3. famsup yes Fedu3
		4. schoolsup no	4. school GP
		5. freetime5 goout2	5.romantic yes freetime5
		6. freetime1	6. paid yes studytime3
		7. Dalc4	7. school MS
		8. romantic yes freetime3	8. freetime5 goout5
		9. famsup yes Mjob teacher	9. freetime2 goout1
		10. failures2	10. higher no
Random Forest	Classes(A-E)	1. absences	1. absences
		2. age2	2. age2
		3. Guard mo age	3. failures0
		4. age	4. age
		5. failures0	5. Guard mo age
		6. romantic yes age	6. romantic yes age
		7. Mjob other	7. school GP
		8. reason course	8. failures1
		9. health5	9. reason course
		10.famrel4	10. sex M
Random Forest	Classes(P/F)	1. absences	1.failures0
		2. failures0	2. school MS
		3. age2	3. higher yes
		4. age	4. absences
		5. Guard mo age	5. failures1
		6. failures 2	6. school GP
		7. romantic yes age	7. higher no
		8. schoolsup yes	8. Guard mo age
		9. romantic yes freetime3	9. age
		10. failures1	10. age2
Lasso Reg	Classes(num)	1. failures0	1. failures0
		2. higher yes Medu4	2. school GP
		3. Mjob services	3. schoolsup no
		4. studytime3	4. higher yes Medu4
		5. goout2	5. sex F
		6. famsize GT3	6. goout5
		7. reason course	7. Fedu1
		8. sex F	8. studytime1
		9. freetime3	9. health5
		10.romantic yes freetime3	10. higher no

### Stage 2: post-double-LASSO for the control variables

To draw statistical inferences and even to make causal statements, one should properly address different aspects of endogeneity embedded in each explanatory variable. One of the major threats is omitted variable bias, and a classical challenge in the big data era is that a large set of candidate control variables is available, but there is minimal prior knowledge on which subset of the control variables should be used. Mathematically, there are two competing forces. On the one hand, if any confounding factor is missing from the model, the linear regression estimators will be biased and inconsistent. On the other hand, if too many controls are included, the correlation among them will cause a close-to-perfect-multicollinearity situation, which undermines the stability of the point estimates.

The conventional approach is to report regression results for several different sets of controls and show that the parameter of interest is insensitive to changes in the set of control variables. This strategy relies on existing theories to offer guidance about which variables to use. However, when the setup of the problem is new, variable selection is usually arbitrary. Inspired by the rapid development and popularity of various dimension reduction methods in machine learning, in a seminal paper, Belloni et al.^41^ proposed a post-double-selection procedure to select the set of control variables at the discretion of the model in lieu of arbitrary handpicking. While many previous studies have utilized this intuitive idea of using machine learning to select control variables before running a linear regression, their approaches usually suffer from two sources of bias. The first bias is due to imperfect variable selection—i.e., variables at the margin of the choice are retained or dropped arbitrarily. The second is the single-selection bias that arises if the selection is conducted only once with the outcome variable on the left-hand side of the model.

Belloni et al.^41^ provided rigorous mathematical proofs along with simulation studies to demon strate the severity of the aforementioned two types of bias and demonstrate the validity of a so-called post-double-selection procedure. In essence, the procedure is to perform variable selection twice using an appropriate dimension reduction algorithm. When LASSO is the chosen method, it is called post-double-LASSO. The procedure works in the following way. First, this study runs a LASSO of *y* on a set of candidate control variables to select a subset of predictors for *y*. Then, the research runs LASSO for the explanatory variable of interest, *d*, on the set of candidate control variables to select a set of predictors for *d*. Last, a linear regression of *y* on *d* is implemented and take the union of the sets of the regressors selected in the two LASSO runs; the inference is simply the usual Heteroskedasticity robust inference in this regression.

One could make sense of this procedure from the perspective of omitted variable bias. In linear regression, omitted variable bias occurs when a confounder—i.e., a variable that is correlated with both *y* and *d*—is excluded from the model. From this standpoint, the first LASSO filters regressors that are associated with *y*, and the second LASSO retains variables that are related to *d*. The union of the two sets, therefore, should include all potential confounders given the data at hand.

Table 5 shows the variables that are selected by the post-double-LASSO procedure. Many variables are commonly selected, for example, the address of a student (rural vs. urban), who is the guardian, whether the student is engaged in a romantic relationship, the reason for the course choice, etc. Due to the collinearity among variables, in the three illustrative linear regressions in the next section, a subset of the post-double-LASSO variables will be used as the control variables.

**Table 5:** Stage 2: post-double-LASSO (Math and Portuguese).

	Method	Y: G3					
2.1	Lasso Reg	1. romantic	2. address	3. goout	4. internet	5. schoolsup	6. reason
		7. health	8. famsize	9. traveltime	10. paid	11. age	12. freetime
		13. sex, 19. activities	14. famsup, 20. guardian	15. Dalc	16. Walc	17. school	18.famrel

	Method	Y: Medu					
2.2 Case #1	Lasso Reg	1.Pstatus	2. Walc	3. guardian	4. reason	5. traveltime	6. famrel
		7. health	8. schoolsup	9. famsize	10. romantic	11. health	12. age
		13. address 19. famsup	14. gout 20. nursery	15. activities, 21. internet	16. sex 22. school	17. paid	18. freetime

	Method	Y: Fedu					
2.2 Case #1	Lasso Reg	1. traveltime	2. health	3. guardian	4. internet	5. goout	6. activities
		7. age	8. nursery	9. famsup	10. address	11. school	12. sex

	Method	Y: Higher					
2.2 Case #2	Lasso Reg	1. goout	2. famrel	3. traveltime	4. age	5. reason	6. health
		7. schoolsup	8. freetime	9. Dalc	10. guardian	11. school	12. famsup
		13. romantic, 19. Walc	14. internet, 20. address	15. activities, 21. paid	16. sex 22. famsize	17. nursery	18. Pstatus

	Method	Y: Absences					
2.2 Case #3	Lasso Reg	1. age	2. Pstatus	3.Walc	4. school	5. Dalc	

### Stage 3: inference based on OLS regression

In this section, this paper will conduct three sets of linear regressions to answer the questions posted at the end of Section "Stage 1: machine learning". The main challenge is the endogeneity embedded in each explanatory variable, ranging from unobserved confounders to reverse causality. Along the way, the research will demonstrate how the control variables selected in the second stage help in modeling the regressions and how sensitivity analysis could be employed to enhance the credibility of the regression coefficients under omitted variable bias.

#### Case # 1. Parents’ occupation and educational level

In theory, a professional occupation and a decent educational degree of the parents would help to build study environment conducive to better academic outcomes for the student.

To gauge which factor is more influential, it is tempting to place all one-hot indicators of occupation type and educational level on the right-hand side of the linear regression. Nevertheless, collinearity among the variables would impede stable point estimates. That said, the question is handled in two steps: first, this paper regresses the course grades on the occupation type and educational level for each part of the parents. The most significant factor from each parent is retained and combined in a follow-up regression to see which combination of mother-father and occupation-education plays the most important role in boosting the course performance of the student.

The first-step regression takes the following form:

1 $$Grade3_{i} = \beta_{0} + \beta_{1} job_{i} + \beta_{2} edu_{i} + \gamma Controls_{i} + \varepsilon_{i} ,$$

where *Grade*3_*i*_ is the course grade of student *i* in the 3rd observational period; *job*_*i*_ is a categorical variable indicating four types of parental occupations—teacher, health industry, service sector, and staying at home—and other; *edu*_*i*_ is also categorical, indicating five types of educational level, 0 (none), 1 (primary education (up to 4th grade)), 2 (5th to 9th grade), 3 (secondary education) and 4 (higher education); and *Controls*_*i*_ stands for a collection of control variables informed by the results in stage 2.

The chief threat to the regression method above is omitted variable bias due to unobserved student ability. It has been established in many existing studies that parental profession and educational background are positively correlated with the ability of the children. Hence, the omission of student ability would inflate the point estimate.

The traditional approach is to either use fixed effects in a panel regression to absorb the impact of ability or include a proxy for student ability. Due to data availability, neither is feasible in the UCI dataset. Notably, while the course grades in the preceding observational periods could shed light on student ability, they should not be used as the proxy because they are also the outcomes of the explanatory variables, making them bad controls, as suggested in^42^.

Recent advances in econometrics provide an alternative—the sensitivity analysis introduced in^43^. The idea is to add a hypothetical variable to the linear regression that is contrived to have an impact at different levels of magnitude than the existing variables. By varying the variable to be compared, one can see how the point estimate and its *t*-statistics would change under different scenarios. In this way, despite not controlling for the confounders, one is able to tell whether, in the worst case, the error in the point estimates of the core explanatory variables would become insignificantly different from zero.

Tables 6 and 7 show the estimated coefficients in Eq. (1) under different specifications of the control variables. Note that while the results in stage 2 suggest a larger set of control variables, the research experiments on the subsets and demonstrate the robustness of the regression coefficients when the subset of controls is both reflective and of a reasonably low variance inflation factor (VIF).

**Table 6:** Effect of *Medu* and *Mjob* on grades (G1, G2 and G3) in Eq. (1) of case #1 in Math and Portuguese.

	(1)	(2)	(3)	(4)	(5)	(6)	(7)	(8)	(9)	(10)	(11)	(12)	(13)	(14)
	*g*_*3*_^*M*^	*g*_*3*_^*M*^	*g*_*3*_^*M*^	*g*_*3*_^*M*^	*g*_*3*_^*M*^	*g*_*1*_^*M*^	*g*_*2*_^*M*^	*g*_*3*_^*P*^	*g*_*3*_^*P*^	*g*_*3*_^*P*^	*g*_*3*_^*P*^	*g*_*3*_^*P*^	*g*_*1*_^*P*^	*g*_*2*_^*P*^
Mjob (tea.)	1.27* (0.68)	0.99 (0.66)	0.99 (0.66)	− 0.61 (0.79)	− 0.60 (0.81)	0.13 (0.65)	− 0.34 (0.67)	1.46*** (0.44)	1.59*** (0.46)	1.33*** (0.46)	0.44 (0.56)	0.51 (0.56)	0.34 (0.49)	0.13 (0.49)
Mjob (hea.)	2.36*** (0.81)	2.18*** (0.80)	2.20*** (0.80)	0.98 (0.87)	0.98 (0.87)	1.14* (0.67)	1.09* (0.66)	1.38*** (0.47)	1.36*** (0.45)	1.15*** (0.42)	0.48 (0.48)	0.44 (0.48)	0.16 (0.44)	0.16 (0.44)
Mjob (ser.)	1.23** (0.60)	1.19** (0.58)	1.21** (0.58)	0.87 (0.58)	0.88 (0.59)	1.01** (0.44)	0.80 (0.50)	0.47 (0.33)	0.51 (0.32)	0.29 (0.31)	0.10 (0.32)	0.098 (0.32)	− 0.015 (0.27)	− 0.10 (0.30)
Mjob (at home)	− 0.73 (0.73)	− 0.51 (0.75)	− 0.52 (0.75)	− 0.016 (0.79)	− 0.014 (0.80)	0.74 (0.52)	0.065 (0.63)	− 0.66* (0.34)	− 0.75** (0.33)	− 0.47 (0.33)	− 0.30 (0.32)	− 0.27 (0.32)	− 0.48* (0.29)	− 0.37 (0.29)
Age		− 0.50*** (0.18)	− 0.55*** (0.20)	− 0.49** (0.20)	− 0.48** (0.22)	− 0.085 (0.17)	− 0.31* (0.18)		− 0.23** (0.10)	− 0.18* (0.098)	− 0.17* (0.098)	− 0.11 (0.11)	− 0.22** (0.089)	− 0.078 (0.095)
Gender (female)		− 0.77* (0.45)	− 0.77* (0.45)	− 0.84* (0.44)	− 0.83* (0.45)	− 0.57* (0.33)	− 0.58 (0.38)		1.11*** (0.26)	1.18*** (0.25)	1.18*** (0.25)	1.20*** (0.25)	0.92*** (0.20)	0.94*** (0.22)
School (GP)			− 0.48 (0.74)	− 0.67 (0.70)	− 0.65 (0.70)	− 0.27 (0.55)	− 0.39 (0.56)			1.74*** (0.29)	1.64*** (0.30)	1.69*** (0.30)	1.44*** (0.24)	1.41*** (0.26)
Medu (5th-9th.)				0.83 (0.76)	0.83 (0.77)	0.81 (0.50)	1.01* (0.56)				0.32 (0.35)	0.32 (0.35)	0.27 (0.27)	0.29 (0.31)
Medu (sec.)				1.28 (0.79)	1.27 (0.80)	0.78 (0.54)	0.99 (0.64)				0.52 (0.37)	0.52 (0.37)	0.35 (0.31)	0.56* (0.33)
Medu (hig.)				2.80*** (0.85)	2.79*** (0.85)	2.08*** (0.65)	2.41*** (0.68)				1.36*** (0.47)	1.35*** (0.48)	1.23*** (0.43)	1.55*** (0.39)
Gua. (mot.)					0.16 (0.98)	− 0.34 (0.68)	− 0.11 (0.79)					0.51 (0.48)	0.68* (0.39)	0.78* (0.45)
Gua. (fat.)					0.22 (1.07)	− 0.10 (0.74)	0.27 (0.86)					1.05* (0.55)	1.23*** (0.43)	1.28** (0.51)
Adjusted *R*^2^	0.029	0.051	0.050	0.069	0.065	0.052	0.064	0.038	0.070	0.130	0.140	0.144	0.171	0.143
Observations	392	392	392	392	392	392	392	643	643	643	643	643	643	643

Standard errors in parentheses.

∗*p* < 0.1;  ∗∗*p* < 0.05; ∗∗∗*p* < 0.01.

**Table 7:** Effect of *Fedu* and *Fjob* on grades (G1, G2 and G3) in Eq. (1) of case #1 in Math and Portuguese.

	(1)	(2)	(3)	(4)	(5)	(6)	(7)	(8)	(9)	(10)	(11)	(12)	(13)	(14)
	*g*_*3*_^*M*^	*g*_*3*_^*M*^	*g*_*3*_^*M*^	*g*_*3*_^*M*^	*g*_*3*_^*M*^	*g*_*1*_^*M*^	*g*_*2*_^*M*^	*g*_*3*_^*P*^	*g*_*3*_^*P*^	*g*_*3*_^*P*^	*g*_*3*_^*P*^	*g*_*3*_^*P*^	*g*_*1*_^*P*^	*g*_*2*_^*P*^
Fjob (tea.)	1.80* (1.05)	1.56 (1.05)	1.56 (1.05)	0.98 (1.15)	0.98 (1.16)	1.60* (0.83)	0.83 (1.04)	1.69*** (0.58)	1.71*** (0.60)	1.45** (0.63)	0.75 (0.66)	0.64 (0.65)	0.80 (0.66)	0.78 (0.57)
Fjob (hea.)	1.44* (0.81)	1.23 (0.84)	1.24 (0.84)	0.85 (0.92)	0.87 (0.94)	0.45 (0.77)	0.39 (0.87)	0.68 (0.66)	0.48 (0.64)	0.33 (0.60)	− 0.29 (0.63)	− 0.40 (0.63)	− 0.29 (0.59)	− 0.27 (0.60)
Fjob (ser.)	0.13 (0.53)	0.17 (0.52)	0.15 (0.52)	0.0075 (0.52)	0.022 (0.52)	0.15 (0.39)	0.35 (0.40)	− 0.26 (0.30)	− 0.24 (0.30)	− 0.071 (0.29)	− 0.19 (0.29)	− 0.26 (0.28)	− 0.093 (0.23)	− 0.20 (0.25)
Fjob (at home)	− 0.017 (1.21)	0.38 (1.24)	0.36 (1.25)	0.28 (1.27)	0.28 (1.27)	1.14 (0.89)	0.31 (1.13)	− 0.50 (0.53)	− 0.55 (0.53)	− 0.064 (0.48)	− 0.0080 (0.47)	− 0.051 (0.46)	− 0.61 (0.41)	− 0.41 (0.45)
Age		− 0.53*** (0.18)	− 0.56*** (0.20)	− 0.50** (0.21)	− 0.48** (0.23)	− 0.072 (0.16)	− 0.31 (0.19)		− 0.26** (0.10)	− 0.21** (0.10)	− 0.18* (0.097)	− 0.11 (0.11)	− 0.21** (0.091)	− 0.077 (0.096)
Gender (female)		− 0.90** (0.45)	− 0.90** (0.45)	− 0.85* (0.45)	− 0.85* (0.46)	− 0.55 (0.34)	− 0.57 (0.38)		0.96*** (0.26)	1.10*** (0.25)	1.16*** (0.25)	1.18*** (0.25)	0.90*** (0.20)	0.91*** (0.22)
School (GP)			− 0.31 (0.74)	− 0.36 (0.72)	− 0.35 (0.73)	− 0.031 (0.55)	− 0.054 (0.57)			1.91*** (0.29)	1.73*** (0.29)	1.79*** (0.29)	1.53*** (0.23)	1.47*** (0.26)
Fedu (5th-9th.)				0.81 (0.67)	0.81 (0.68)	1.20** (0.48)	1.26** (0.53)				0.71** (0.33)	0.67** (0.33)	0.63** (0.26)	0.54* (0.30)
Fedu (sec.)				1.21* (0.65)	1.22* (0.66)	0.94* (0.49)	1.10** (0.56)				1.13*** (0.32)	1.08*** (0.32)	0.79*** (0.28)	0.96*** (0.28)
Fedu (hig.)				1.45* (0.75)	1.45* (0.75)	1.64*** (0.54)	1.61*** (0.62)				1.46*** (0.39)	1.44*** (0.39)	1.12*** (0.34)	1.32*** (0.36)
Gua. (mot.)					0.26 (0.97)	− 0.13 (0.67)	0.079 (0.78)					0.81* (0.47)	0.94** (0.37)	1.03** (0.44)
Gua. (fat.)					0.17 (1.06)	− 0.12 (0.74)	0.30 (0.85)					1.22** (0.53)	1.39*** (0.42)	1.41*** (0.50)
Adjusted *R*^2^	0.003	0.030	0.028	0.032	0.027	0.044	0.031	0.014	0.041	0.116	0.134	0.138	0.159	0.131
Observations	393	393	393	393	393	393	393	642	642	642	642	642	642	642

Standard errors in parentheses.

*p < 0.1; **p < 0.05; ***p < 0.01.

The main results of interest are *g*_*3*_^*M*^ and *g*_*3*_^*P*^—the course grades in the 3rd observational period for Math and Portuguese. For robustness, the estimates are shown for these two subjects in the first two periods. Note that in all regressions, observations where parental education is below elementary school are excluded, as they account for less than 1% of the entire sample and can be regarded as outliers.

Using an occupation of other as the benchmark, while in some scenarios (e.g., the mother’s job being health care and the father’s being teacher) the estimates are significantly positive, their effects become indistinguishable from zero when indicators for the educational levels are added and the full set of control variables are included.

In contrast, educational level exhibits a more stable impact on course grades for both parents and both subjects. It is shown that compared to an elementary school degree, if either of the parents has a higher degree, the course grades of their children are on average approximately 1.5 points higher than others. Notably, the impact of a higher degree of the mother is larger in Math than in Portuguese, despite there being no substantial heterogeneity in the impact of the father’s degree.

**Fig. 5:**
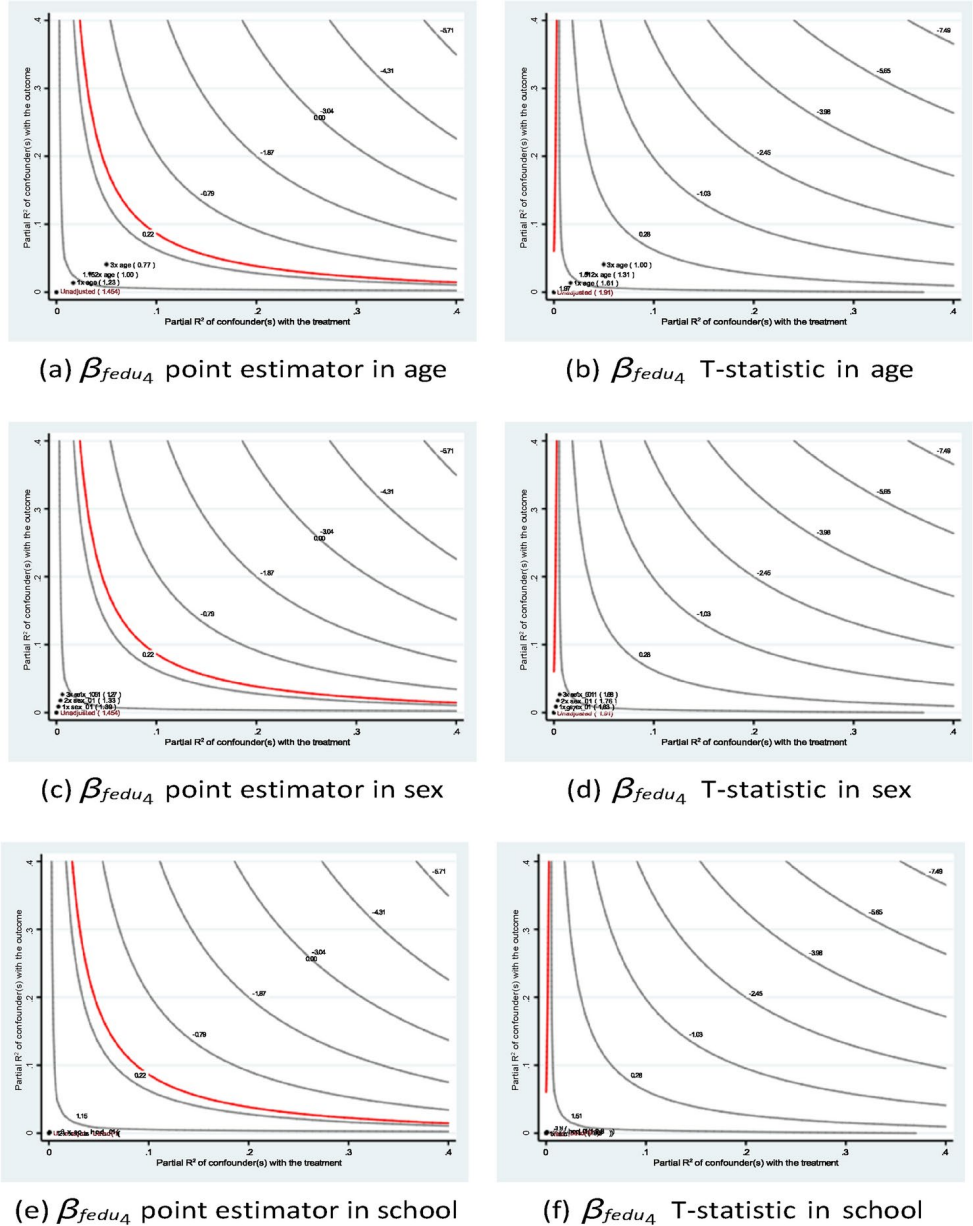
Sensitivity analysis of *fedu*4 in Eq. (1) of case #1 in Math.

**Fig. 6:**
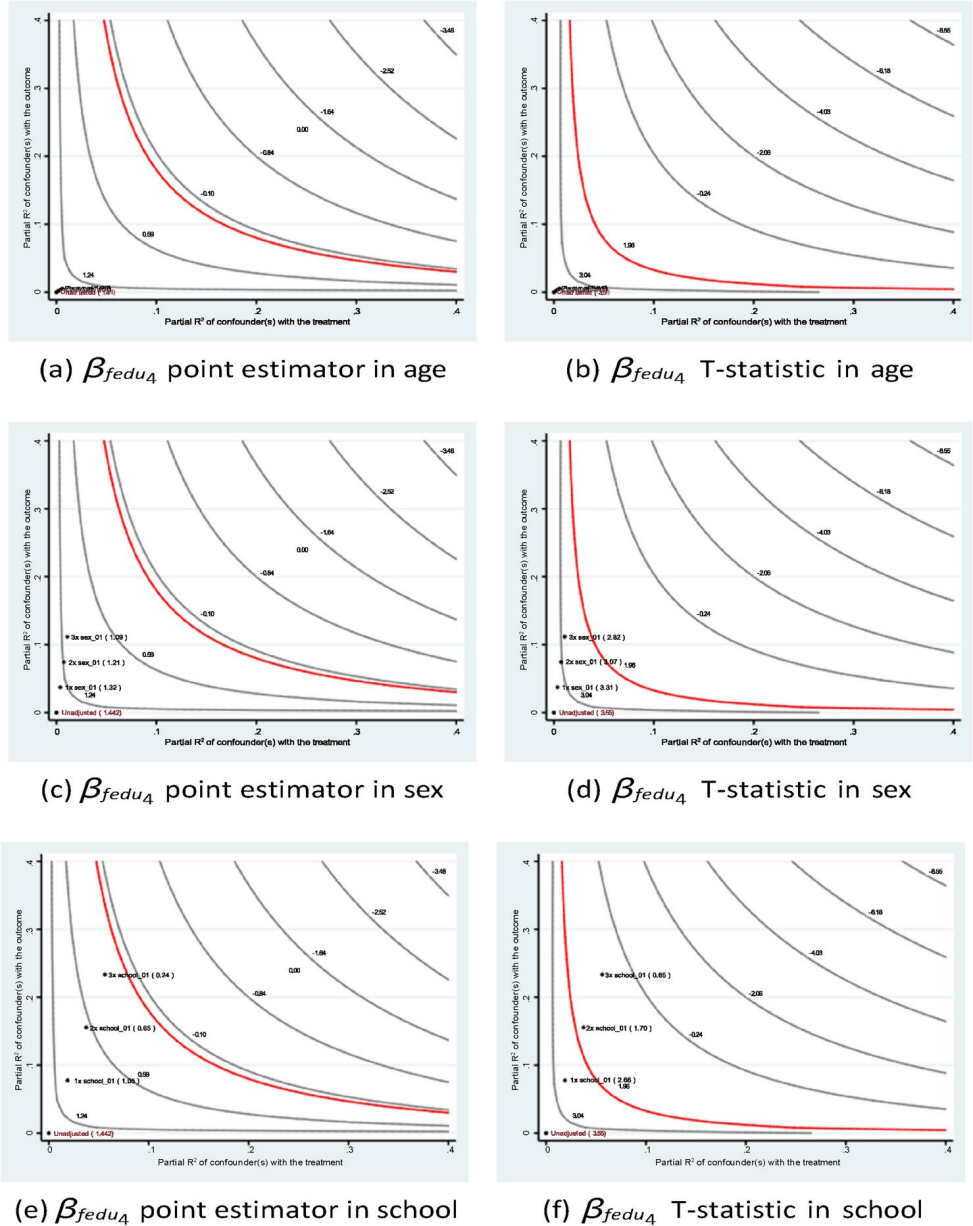
Sensitivity analysis of *fedu*4 in Eq. (1) of case #1 in Portuguese.

Figures 5 and 6 demonstrate the stability of the estimates of the parents’ educational levels under different hypothetical scenarios. Consistent with the qualitative argument, the inclusion of a hypothetical variable that has an impact at different levels of magnitude than existing variables would lead to a lower point estimate in terms of absolute value. In all setups, the *t*-statistics are greater than the critical value of 1*.*96.

These preliminary results on the relative importance of occupation and educational background motivate the following regression to further investigate the contribution of the parents:

2 $$Grade3_{i} = \beta_{0} + \beta_{1} medu_{i} + \beta_{2} fedu_{i} + \gamma Controls_{i} + \varepsilon_{i} ,$$

where the explanatory variables are *medu*_*i*_ and *fedu*_*i*_, with the same definitions as in Eq. (1).

Equation (2) shares the same endogeneity concerns with the previous regression model in terms of unobserved student ability. Therefore, this paper includes the same set of control variables and sensitivity analyses to demonstrate the validity of the estimated coefficients. Additionally, note that students who have at least one parent with an educational level below elementary school are dropped from the estimation to obtain results comparable to those in Eq. (1).

Table 8 exhibits the regression results under different specifications of the controls and for different outcomes of interest. It turns out that the mother having a higher degree is significantly positive in all regression settings, and the father’s educational contribution now becomes negligible. Moreover, the magnitude of the impact is similar to the results of Eq. (1), where the impact is higher for Math and lower for Portuguese.

**Table 8:** Effect of *medu* and *fedu* on grades (G1, G2 and G3) in Eq. (2) of case #1 in Math and Portuguese.

	(1)	(2)	(3)	(4)	(5)	(6)	(7)	(8)	(9)	(10)	(11)	(12)
	*g*_*3*_^*M*^	*g*_*3*_^*M*^	*g*_*3*_^*M*^	*g*_*3*_^*M*^	*g*_*1*_^*M*^	*g*_*2*_^*M*^	*g*_*3*_^*P*^	*g*_*3*_^*P*^	*g*_*3*_^*P*^	*g*_*3*_^*P*^	*g*_1_^*P*^	*g*_2_^*P*^
Medu (5th-9th.)	1.07 (0.78)	0.99 (0.78)	1.11 (0.78)	1.12 (0.78)	0.67 (0.54)	1.08* (0.61)	0.71** (0.36)	0.70** (0.35)	0.29 (0.35)	0.30 (0.35)	0.29 (0.28)	0.29 (0.30)
Medu (sec.)	1.64** (0.81)	1.57* (0.81)	1.68** (0.80)	1.70** (0.82)	0.67 (0.59)	1.22* (0.67)	0.79* (0.42)	0.88** (0.41)	0.42 (0.40)	0.46 (0.40)	0.38 (0.34)	0.49 (0.37)
Medu (hig.)	3.07*** (0.85)	2.84*** (0.86)	2.95*** (0.86)	2.99*** (0.90)	1.71*** (0.63)	2.49*** (0.74)	1.80*** (0.48)	1.89*** (0.48)	1.43*** (0.49)	1.47*** (0.49)	1.43*** (0.38)	1.54*** (0.41)
Fedu (5th-9th.)	0.32 (0.72)	0.11 (0.71)	0.098 (0.72)	0.087 (0.72)	0.78 (0.52)	0.66 (0.59)	0.48 (0.36)	0.46 (0.35)	0.48 (0.33)	0.43 (0.33)	0.40 (0.27)	0.30 (0.30)
Fedu (sec.)	0.18 (0.74)	0.0094 (0.73)	− 0.0042 (0.73)	− 0.046 (0.75)	0.24 (0.56)	0.13 (0.64)	0.77** (0.39)	0.75** (0.37)	0.61* (0.36)	0.53 (0.36)	0.30 (0.32)	0.39 (0.33)
Fedu (hig.)	0.29 (0.88)	0.067 (0.89)	0.039 (0.89)	− 0.0086 (0.91)	1.06 (0.65)	0.40 (0.79)	0.85* (0.48)	0.83* (0.47)	0.69 (0.45)	0.61 (0.45)	0.38 (0.40)	0.50 (0.41)
Age		− 0.44** (0.18)	− 0.50** (0.20)	− 0.48** (0.22)	− 0.050 (0.16)	− 0.30 (0.18)		− 0.20** (0.10)	− 0.16* (0.098)	− 0.10 (0.11)	− 0.20** (0.091)	− 0.067 (0.097)
Gender (female)		− 0.75* (0.45)	− 0.75* (0.45)	− 0.73 (0.45)	− 0.48 (0.33)	− 0.48 (0.38)		1.12*** (0.26)	1.21*** (0.25)	1.23*** (0.25)	0.93*** (0.20)	0.97*** (0.22)
School (GP)			− 0.64 (0.70)	− 0.62 (0.70)	− 0.18 (0.56)	− 0.34 (0.57)			1.66*** (0.30)	1.72*** (0.30)	1.50*** (0.23)	1.42*** (0.26)
Gua. (mot.)				0.041 (0.99)	− 0.32 (0.68)	− 0.17 (0.80)				1.14** (0.54)	1.32*** (0.43)	1.34*** (0.51)
Gua. (fat.)				0.25 (1.08)	− 0.11 (0.75)	0.34 (0.86)				0.64 (0.47)	0.77** (0.38)	0.86* (0.45)
Adjusted *R*^2^	0.046	0.062	0.061	0.057	0.049	0.059	0.063	0.094	0.148	0.152	0.175	0.149
Observations	390	390	390	390	390	390	637	637	637	637	637	637

Standard errors in parentheses.

*p < 0.1; **p < 0.05; ***p < 0.01.

**Fig. 7:**
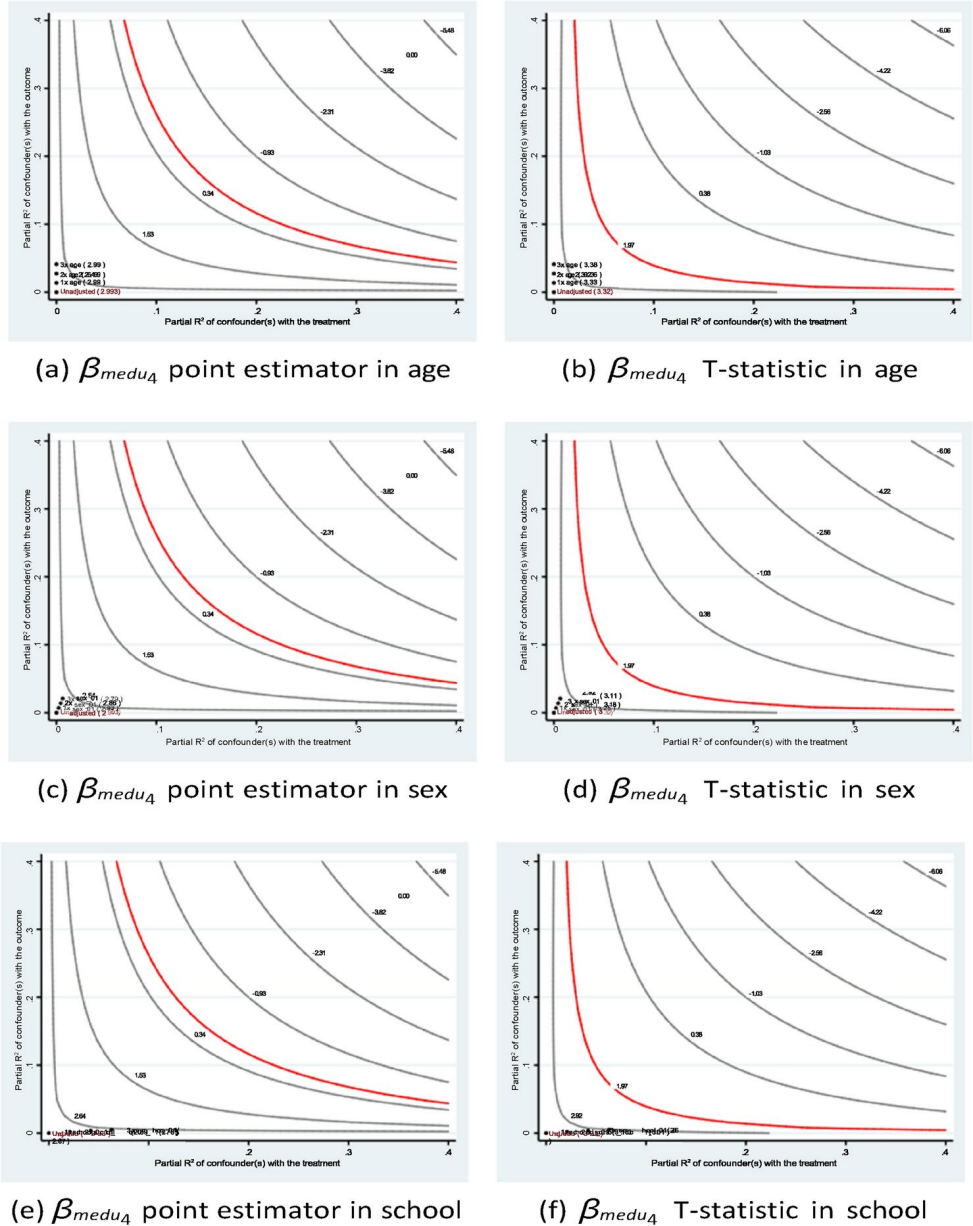
Sensitivity analysis of *medu*4 in Eq. (1) of case #1 in Math.

**Fig. 8:**
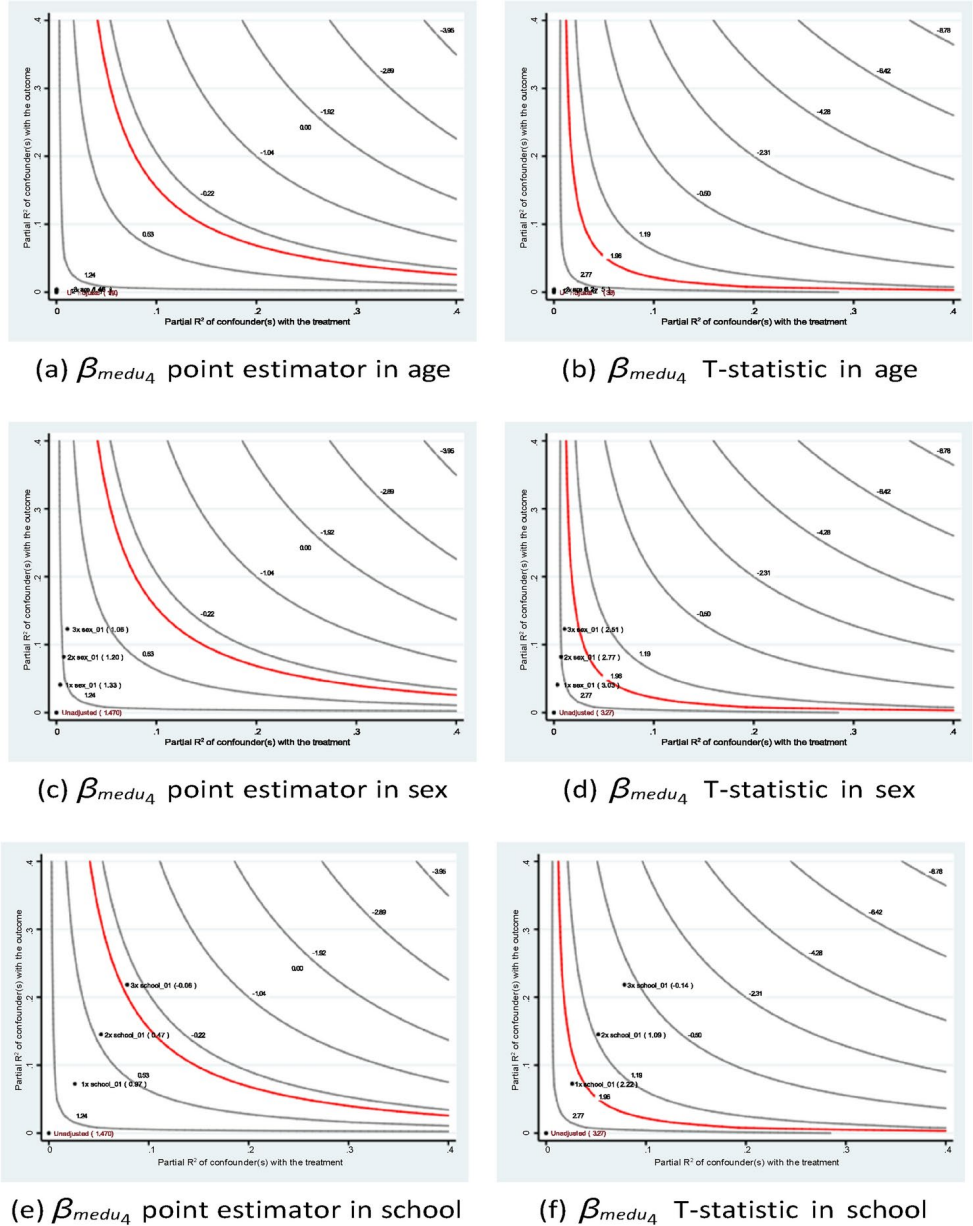
Sensitivity analysis of *medu*4 in Eq. (1) of case #1 in Portuguese.

The sensitivity analysis results shown in Figs. 7 and 8 confirm the stability of the estimates of the father’s education. In short, the research founds a similar pattern of academic contributions from the parents’ educational backgrounds to those found in many existing studies. For instance, Özyıldırım^44^ conducted a meta-analysis of 37 studies involving over 45,000 students to examine the impact of parental involvement on students’ academic motivation. The analysis found that parental involvement has a small but statistically significant overall effect on academic motivation. These findings suggest that policy interventions aimed at improving educational accessibility not only benefit current students but may also positively influence future generations.

However, technically, my work differs by adopting a model-driven approach in which the explanatory variables are first found by machine learning algorithms and the corresponding control variables are selected as a result of post-double-LASSO.

#### Case # 2. Intention of obtaining a higher degree

A considerable proportion of the students in the UCI dataset declared in the survey their intention to pursue a higher degree after graduation. In this section, this paper investigates whether this aspiration leads to better course performance. The following linear regression model is estimated:

3 $$Grade{3}_{i} = \beta_{0} + \beta_{{1}} Higher_{i} + \gamma Controls_{i} + \varepsilon_{i} ,$$

where *Higher*_*i*_ is a binary value taking the value 1 if the student answers yes in the survey and *Controls*_*i*_ stands for a subset of the control variables, as suggested by the second stage.

There are two major empirical threats to Eq. (3). First, similar to the previous case, the unobserved student ability is a confounding factor that is excluded from the model. Presumably, a student with greater talent would prefer additional education and obtain a better grade, which would cause an upward bias when estimating *β*_1_. Second, the description of the dataset does not make the timing of the survey clear; hence, it is unknown whether the indicated intention of further education was declared before or after the students knew their latest grade. In the latter scenario, there would be reverse causality from *Grade*3_*i*_ to *Higher*_*i*_.

Unfortunately, due to the lack of a valid IV for *Higher*_*i*_, this research can only evaluate, to some extent, the association between the two variables. To do so, the same sensitivity analysis is applied to gauge the stability of *β*^ˆ^1 when hypothetical variables are added into the regression.

**Fig. 9:**
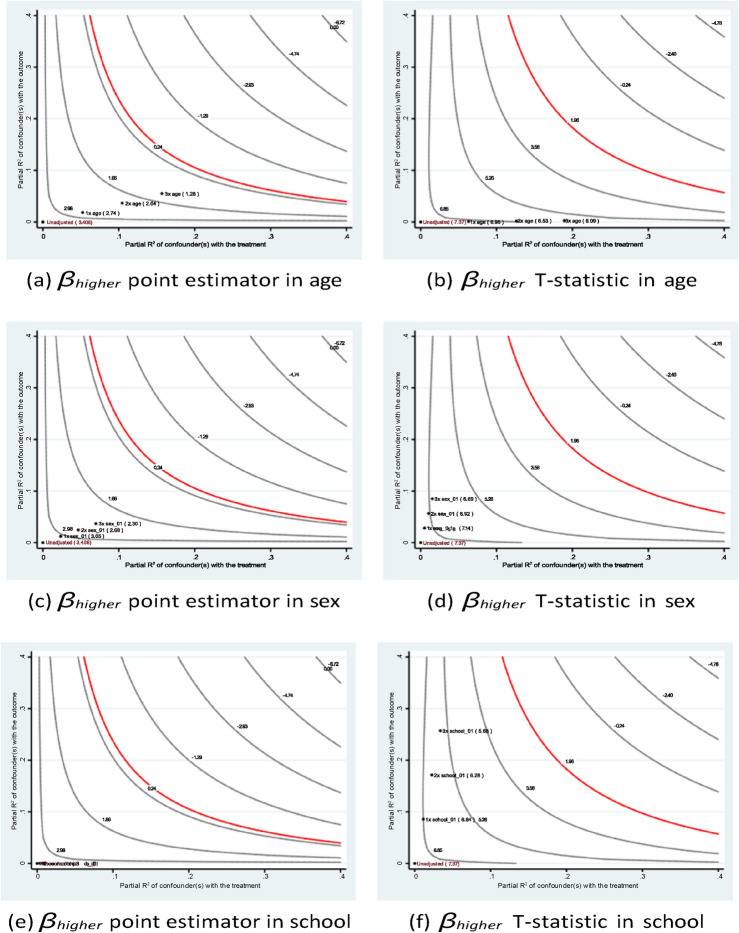
Sensitivity analysis of *higher* in Eq. (3) of case #2 in Math.

**Fig. 10:**
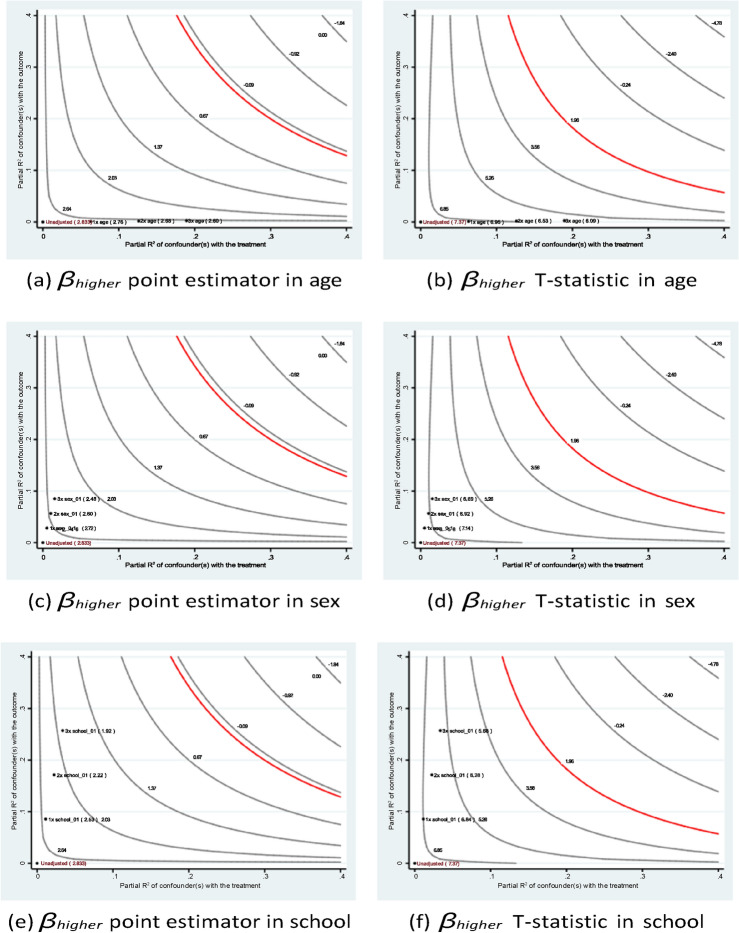
Sensitivity analysis of *higher* in Eq. (3) of case #2 in Portuguese.

Table 9 shows the estimated coefficients of Eq. (3) and Fig. 9 as well as Fig. 10 demonstrate the robustness of the strong positive estimates of *β*^ˆ^1 when hypothetical variables of different levels of impact are added to the regression. Due to collinearity among the candidate control variables, this paper includes only a subset of the variables that are reflective and have substantial variation as suggested by post-double-LASSO. It is shown that the students who intend to pursue a higher degree on average score three more points in Math and two more in Portuguese than other students. The magnitude is high given that most grades cluster between 8 and 15.

**Table 9:** Effect of *Higher*_*i*_ on grades (G1, G2 and G3) of case #2 in Math and Portuguese.

	(1)	(2)	(3)	(4	(5)	(6)	(7)	(8)	(9)	(10)	(11)	(12)	(13)	(14)	(15)	(16)	(17)	(18)
	*g*_*3*_^*M*^	*g*_*3*_^*M*^	*g*_*3*_^*M*^	*g*_*3*_^*M*^	*g*_*3*_^*M*^	*g*_*3*_^*M*^	*g*_*3*_^*M*^	*g*_*1*_^*M*^	_*g*_ *2* ^*M*^	*g*_*3*_^*P*^	*g*_*3*_^*P*^	*g*_*3*_^*P*^	*g*_*3*_^*P*^	*g*_*3*_^*P*^	*g*_*3*_^*P*^	*g*_*3*_^*P*^	*g*_*1*_^*P*^	*g*_*2*_^*P*^
Higher (yes)	3.81*** (1.08)	3.68*** (1.15)	3.67*** (1.15)	3.46*** (1.17)	3.37 (1.16)	3.44*** (1.17)	3.41*** (1.19)	2.81*** (0.69)	2.90*** (0.80)	3.48*** (0.38)	3.34*** (0.39)	2.99*** (0.40)	2.85*** (0.39)	2.80*** (0.38)	2.81*** (0.38)	2.83*** (0.39)	2.47*** (0.26)	2.61*** (0.33)
Age		− 0.43** (0.18)	− 0.45** (0.20)	− 0.38* (0.20)	− 0.40* (0.20)	− 0.41** (0.20)	− 0.50** (0.21)	− 0.16 (0.14)	− 0.35** (0.17)		− 0.071 (0.098)	− 0.038 (0.094)	− 0.0029 (0.097)	− 0.0020 (0.097)	− 0.0059 (0.096)	− 0.066 (0.096)	− 0.22*** (0.084)	− 0.052 (0.084)
Gender (female)		− 1.16*** (0.45)	− 1.16** (0.45)	− 1.23*** (0.44)	− 1.19*** (0.44)	− 1.11** (0.45)	− 0.98** (0.46)	− 0.55* (0.33)	− 0.70* (0.37)		0.73*** (0.25)	0.88*** (0.24)	0.85*** (0.24)	0.86*** (0.24)	0.88*** (0.24)	0.99*** (0.24)	0.77*** (0.19)	0.76*** (0.21)
School (GP)			− 0.11 (0.74)	0.011 (0.75)	− 0.027 (0.75)	0.083 (0.75)	0.14 (0.76)	0.45 (0.59)	0.41 (0.59)			1.73*** (0.27)	1.67*** (0.27)	1.66*** (0.27)	1.66*** (0.27)	1.77*** (0.27)	1.56*** (0.22)	1.48*** (0.24)
Goout (Low)				0.86 (1.16)	0.82 (1.16)	0.86 (1.16)	0.75 (1.19)	− 0.18 (0.69)	0.28 (0.69)				1.43*** (0.55)	1.37** (0.54)	1.39*** (0.53)	1.31** (0.54)	1.04*** (0.40)	1.34*** (0.48)
Goout (Average)				0.73 (1.14)	0.67 (1.13)	0.71 (1.13)	0.55 (1.16)	− 0.39 (0.67)	− 0.12 (0.68)				1.18** (0.53)	1.10** (0.51)	1.12** (0.51)	1.01** (0.51)	0.89** (0.37)	0.94** (0.46)
Goout (High)				− 0.53 (1.18)	− 0.58 (1.16)	− 0.57 (1.16)	− 0.63 (1.19)	− 1.18* (0.70)	− 1.04 (0.72)				0.87 (0.53)	0.80 (0.51)	0.81 (0.51)	0.73 (0.51)	0.46 (0.40)	0.64 (0.47)
Goout (Very high)				− 0.97 (1.26)	− 1.07 (1.26)	− 1.03 (1.26)	− 1.14 (1.29)	− 1.70** (0.76)	− 1.53* (0.82)				0.34 (0.59)	0.28 (0.57)	0.30 (0.57)	0.19 (0.57)	0.42 (0.39)	0.39 (0.50)
Family rel. (Fair)					0.083 (1.94)	0.091 (1.95)	0.35 (1.93)	1.44 (1.39)	0.75 (1.32)					− 0.16 (0.88)	− 0.16 (0.89)	0.12 (0.91)	0.50 (0.74)	0.039 (0.92)
Family rel. (Good)					− 0.19 (1.54)	− 0.21 (1.57)	− 0.0055 (1.59)	0.37 (1.20)	− 0.39 (1.08)					0.062 (0.74)	0.054 (0.74)	0.25 (0.74)	0.11 (0.60)	0.29 (0.75)
Family rel.(Very good)					0.25 (1.46)	0.28 (1.49)	0.50 (1.51)	0.90 (1.15)	− 0.10 (1.03)					0.94 (0.70)	0.95 (0.70)	1.11 (0.70)	0.73 (0.57)	1.14 (0.72)
Family rel. (Excellent)					0.59 (1.50)	0.56 (1.53)	0.78 (1.54)	0.77 (1.17)	− 0.20 (1.06)					0.43 (0.73)	0.43 (0.73)	0.58 (0.73)	0.40 (0.58)	0.75 (0.74)
Famsup. (yes)						− 0.56 (0.46)	− 0.51 (0.46)	− 0.61* (0.33)	− 0.59 (0.37)						− 0.11 (0.24)	− 0.071 (0.23)	− 0.19 (0.20)	− 0.18 (0.22)
Schoolsup. (yes)							− 1.43*** (0.55)	− 2.25*** (0.40)	− 1.59*** (0.43)							− 1.52*** (0.33)	− 1.43*** (0.34)	− 1.21*** (0.28)
Adjusted R ^2^ Observations	0.031 395	0.058 395	0.055 395	0.070 395	0.063 395	0.064 395	0.072 395	0.102 395	0.079 ^95^	0.109 649	0.119 649	0.181 649	0.195 649	0.204 649	0.203 649	0.222 649	0.230 649	0.208 649

Standard errors in parentheses

*p < 0.1; **p < 0.05; ***p < 0.01

This finding offers an additional perspective on the positive impact of government policies aimed at enhancing access to higher education. For instance, in 2024, the Chinese government outlined a set of refined financial aid policies to both reward outstanding students and support those from economically disadvantaged backgrounds. The number of National Scholarship recipients for both undergraduate and graduate students has doubled, accompanied by an increase in scholarship amounts. Such policies are likely to motivate high school students, particularly those facing financial hardship, to work harder, thereby contributing to improved educational outcomes at the K-12 level.

#### Case # 3. Class absences

The other explanatory variable that is highlighted three times by machine learning algorithms in the first stage is the number of class absences. In this section, this work studies the causal effect of absence on academic performance. While it might be apparent that more absences would drive down course grades, the goal of this case study is to complement the first two stages of the pipeline to demonstrate the procedure of the model-driven causal inference.

The following linear regression is estimated:

4 $$Grade{3}_{i} = \beta_{0} + \beta_{{1}} Absences_{i} + \gamma Controls_{i} + \varepsilon_{i} ,$$

where *Absences*_*i*_ is a numerical value ranging from 0 to 93 and the set of control variables is inherited from the results of post-double-LASSO.

In addition to the potential omitted variable bias induced by the unobserved student ability, another major empirical threat to Eq. (3) above is reverse causality, where absences could be a result of unsatisfactory course grades. Therefore, the study instruments *Absence*_*i*_ by *Goout*_*i*_, which records the frequency of social activities. The IV relevance assumption is met here since interest in social activities might motivate the student to skip classes. The IV exclusion restriction is satisfied, as *Goout*_*i*_ is only selected once in the first-stage results. The variable is still deemed relevant by LASSO regression in stage 1. However, *Goout*_*i*_ is the most appropriate IV for *Absences*_*i*_ given data availability.

Tables 10 and 11 exhibit regression coefficients under different setups of the model. To avoid potential bias in the estimates induced by unusual and large values of *Absences*_*i*_, this research also shows the estimated coefficients of the truncated sample, where only students who missed fewer than 22 or 30 classes are used in estimation. The p values for the underidentification test, which are 0.0571 (G3/G2/G1 <22) and 0.0833 (G3/G2/G1 <30), in Stata (i.e., the test of whether the matrices of all endogenous regressors and IVs are of full column rank). In almost all settings, the test is passed at the 10% confidence level.

**Table 10:** Effect of *Absences* on grades (G1, G2 and G3) of case #3 in Math.

	(1)	(2)	(3)	(4)	(5)	(6)	(7)	(8)	(9)	(10)	(11)	(12)	(13)
	*g*_*3*_^*M*^	*g*_*3*_^*M*^	*g*_*3*_^*M*^	*g*_*3*_^*M*^	*g*_*3*_^*M*^ (< 38)	*g*_*3*_^*M*^ (< 50)	*g*_*3*_^*M*^	*g1*^*M*^ (< 38)	*g1*^*M*^ (< 50)	*g1* ^*M*^	*g2*^*M*^ (< 38)	*g2*^*M*^ (< 50)	*g2* ^*M*^
Absences	0.020	0.041	0.044	0.050*	− 0.99	− 0.87	− 1.27	− 0.86*	− 0.77*	− 0.69	− 0.96	− 0.83	− 0.97
	(0.024)	(0.026)	(0.027)	(0.028)	(0.67)	(0.61)	(1.56)	(0.52)	(0.46)	(0.85)	(0.59)	(0.51)	(1.15)
Age		− 0.61***	− 0.66***	− 0.77***	0.42	0.53	1.32	0.67	0.78	0.79	0.56	0.64	1.01
		(0.19)	(0.21)	(0.21)	(0.84)	(0.90)	(2.42)	(0.65)	(0.69)	(1.33)	(0.72)	(0.75)	(1.78)
Gender (female)		− 0.95**	− 0.95**	− 0.77	− 0.68	− 0.68	0.35	− 0.24	− 0.24	0.30	− 0.37	− 0.38	0.39
		(0.45)	(0.45)	(0.47)	(0.79)	(0.77)	(1.50)	(0.59)	(0.59)	(0.85)	(0.68)	(0.66)	(1.14)
School (GP)			− 0.47	− 0.70	2.45	2.84	5.50	2.57	2.94	3.25	2.57	2.87	4.27
			(0.76)	(0.77)	(2.23)	(2.46)	(6.82)	(1.76)	(1.94)	(3.77)	(1.93)	(2.08)	(4.98)
Extra act. (yes)				0.027	0.059	− 0.24	− 0.17	0.39	0.12	0.26	0.35	0.058	0.18
				(0.47)	(0.79)	(0.77)	(1.14)	(0.60)	(0.60)	(0.63)	(0.69)	(0.66)	(0.85)
Add. (urban)				1.07*	1.10	0.74	− 0.13	0.57	0.25	− 0.12	1.10	0.77	0.17
				(0.58)	(0.93)	(0.99)	(1.66)	(0.70)	(0.77)	(0.94)	(0.82)	(0.87)	(1.23)
Schoolsup. (yes)				− 1.62***	− 0.91	− 0.98	0.026	− 1.75**	− 1.81**	− 1.45	− 1.06	− 1.14	− 0.47
				(0.54)	(1.03)	(0.95)	(2.70)	(0.86)	(0.81)	(1.49)	(0.92)	(0.84)	(2.00)
Famsup. (yes)				− 0.38	0.22	0.061	− 0.027	0.028	− 0.12	− 0.30	0.10	− 0.051	− 0.19
				(0.47)	(0.92)	(0.86)	(1.46)	(0.70)	(0.66)	(0.80)	(0.79)	(0.73)	(1.09)
Adjusted *R*^2^	− 0.001	0.033	0.032	0.046	− 1.740	− 1.567	− 5.000	− 2.136	− 1.978	− 2.560	− 2.091	− 1.813	− 4.026
Observations	395	395	395	395	390	392	395	390	392	395	390	392	395

Standard errors in parentheses.

*p < 0.1; **p < 0.05; ***p < 0.01.

**Table 11:** Effect of *Absence* on grades (G1, G2 and G3) of case #3 in Portuguese.

	(1)	(2)	(3)	(4)	(5)	(6)	(7)	(8)	(9)	(10)	(11)	(12)	(13)
	*g*_*3*_^*P*^	*g*_*3*_^*P*^	*g*_*3*_^*P*^	*g*_*3*_^*P*^	*g*_*3*_^*P*^ (< 22)	*g*_*3*_^*P*^ (< 30)	*g*_*3*_^*P*^	*g*_*1*_^*P*^ (< 22)	*g*_*1*_^*P*^ (< 30)	*g*_*1*_^*P*^	*g*_*2*_^*P*^ (< 22)	*g*_*2*_^*P*^ (< 30)	*g*_*2*_^*P*^
Absences	− 0.064**	− 0.090***	− 0.090***	− 0.097***	− 0.76**	− 0.72**	− 0.67**	− 0.63**	− 0.58**	− 0.56**	− 0.68**	− 0.64**	− 0.61**
	(0.027)	(0.026)	(0.026)	(0.026)	(0.32)	(0.32)	(0.32)	(0.26)	(0.25)	(0.26)	(0.29)	(0.28)	(0.29)
Age		− 0.18*	− 0.17*	− 0.23**	0.17	0.15	0.13	− 0.026	− 0.056	− 0.058	0.15	0.12	0.12
		(0.10)	(0.10)	(0.100)	(0.24)	(0.24)	(0.24)	(0.20)	(0.19)	(0.20)	(0.21)	(0.21)	(0.21)
Gender (female)		1.02***	1.06***	1.15***	0.99***	0.94***	1.06***	0.77***	0.72***	0.82***	0.76***	0.70**	0.80***
		(0.25)	(0.25)	(0.25)	(0.33)	(0.33)	(0.32)	(0.26)	(0.26)	(0.25)	(0.29)	(0.28)	(0.28)
School (GP)		2.12***	1.90***	2.03***	2.99***	3.02***	3.02***	2.57***	2.56***	2.60***	2.61***	2.61***	2.64***
		(0.27)	(0.29)	(0.29)	(0.61)	(0.63)	(0.65)	(0.49)	(0.50)	(0.54)	(0.55)	(0.57)	(0.60)
Extra act. (yes)			0.32	0.29	0.12	0.13	0.15	0.18	0.19	0.21	0.14	0.16	0.17
			(0.24)	(0.24)	(0.33)	(0.32)	(0.32)	(0.26)	(0.25)	(0.25)	(0.29)	(0.29)	(0.28)
Add. (urban)			0.57*	0.54*	0.51	0.57	0.63*	0.34	0.39	0.44	0.41	0.46	0.51
			(0.29)	(0.29)	(0.38)	(0.37)	(0.36)	(0.31)	(0.30)	(0.29)	(0.34)	(0.33)	(0.33)
Schoolsup. (yes)				− 1.55***	− 1.94***	− 1.98***	− 2.05***	− 1.76***	− 1.79***	− 1.86***	− 1.59***	− 1.62***	− 1.70***
				(0.32)	(0.47)	(0.48)	(0.50)	(0.44)	(0.44)	(0.46)	(0.42)	(0.42)	(0.44)
Famsup. (yes)				0.17	0.44	0.39	0.46	0.23	0.18	0.24	0.29	0.24	0.31
				(0.25)	(0.35)	(0.34)	(0.35)	(0.29)	(0.28)	(0.30)	(0.31)	(0.30)	(0.31)
Adjusted *R*^2^	0.007	0.122	0.128	0.146	− 0.520	− 0.496	− 0.504	− 0.358	− 0.306	− 0.372	− 0.475	− 0.424	− 0.481
Observations	649	649	649	649	643	647	649	643	647	649	643	647	649

Standard errors in parentheses.

*p < 0.1; **p < 0.05; ***p < 0.01.

The use of IV allows us to interpret the result as causal. Specifically, it is shown that each additional absence from class leads to a decline of 0*.*6 to 0*.*76 points in Portuguese. However, the impact of absences on course grades in Math is not significant. This might be due to the intrinsic difference between learning linguistics and science, where the former relies more heavily upon class participation, while the latter could be largely self-studied.

This finding aligns with existing research (e.g., see^45^ and more recently^46^) and provides statistical support for the widespread policy of recording attendance across various levels of education worldwide.

## Concluding remarks

A three-stage pipeline is proposed that aims at conducting statistical and causal inference on big data in education when minimal prior knowledge is assumed. The pipeline starts with a screening of all variables and their meaningful transformations to identify potential explanatory variables and moderators. After deciding which explanatory variables are of interest, a post-double-selection procedure is carried out to select the set of control variables. Finally, using linear regression and IVs (whenever possible), this paper describes three typical case studies in education, where different aspects of endogeneity are reviewed and resolved.

Notably, built upon the recent rigorous synthesis between econometrics and machine learning, the pipeline improves on the intuitive approach of combining the two strands of methods in educational studies by introducing a second stage—post-double-LASSO. In addition, sensitivity analysis that has been increasingly popular in econometrics are also implemented in this paper to show the stability of the point estimates under omitted variable bias. While the conclusions drawn in the case studies on stage 3 are not groundbreaking in the literature, both the pipeline and the technique of sensitivity analysis are new and highly adaptive to a much wider spectrum of empirical studies in education.

The proposed three-stage pipeline opens up a wide range of possibilities for future research in educational studies and beyond. Further methodological development can enhance the robustness of the framework, particularly by exploring alternative machine learning techniques for constructing valid IVs in the third stage from existing variables. In education, this framework can be adapted to investigate long-term causal effects on student outcomes, such as career trajectories or social mobility, and the interdisciplinary researches with psychology where a rich set of psychological factors are to be investigated. Moreover, the integration of dynamic panel data models within this pipeline could address time-varying confounders, expanding its applicability to longitudinal studies, as individual and time fixed effects are compatible with each stage of this pipeline.

Lastly, extending this work to account for heterogeneity in treatment effects—particularly across different demographic or socio-economic groups—could provide valuable insights for designing inclusive educational policies. These avenues underscore the potential of the proposed framework to transform empirical research and bridge the gap between theory and executable insights.

## Data Availability

The raw data and materials supporting Figs. 1, 2, Fig. 10, are publicly available at the UC Irvine Machine Learning Repository, http://archive.ics.uci.edu/dataset/320/ student + performance, and none of the experiments was preregistered.
